# Aquaporins influence seed dormancy and germination in response to stress

**DOI:** 10.1111/pce.13561

**Published:** 2019-05-09

**Authors:** Steven Footitt, Rachel Clewes, Mistianne Feeney, William E. Finch‐Savage, Lorenzo Frigerio

**Affiliations:** ^1^ School of Life Sciences University of Warwick Warwickshire CV4 7AL UK

**Keywords:** abscisic acid, aquaporin, dormancy, germination, global warming, tonoplast intrinsic protein, water stress

## Abstract

Aquaporins influence water flow in plants, yet little is known of their involvement in the water‐driven process of seed germination. We therefore investigated their role in seeds in the laboratory and under field and global warming conditions. We mapped the expression of tonoplast intrinsic proteins (TIPs) during dormancy cycling and during germination under normal and water stress conditions. We found that the two key tonoplast aquaporins, TIP3;1 and TIP3;2, which have previously been implicated in water or solute transport, respectively, act antagonistically to modulate the response to abscisic acid, with TIP3;1 being a positive and TIP3;2 a negative regulator. A third isoform, TIP4;1, which is normally expressed upon completion of germination, was found to play an earlier role during water stress. Seed TIPs also contribute to the regulation of depth of primary dormancy and differences in the induction of secondary dormancy during dormancy cycling. Protein and gene expression during annual cycling under field conditions and a global warming scenario further illustrate this role. We propose that the different responses of the seed TIP contribute to mechanisms that influence dormancy status and the timing of germination under variable soil conditions.

## Abstract 2

Aquaporins influence water flow in plants, yet little is known of their involvement in the water‐driven process of seed germination. We therefore investigated their role in seeds, both in the laboratory and field. We mapped the expression of tonoplast intrinsic proteins during germination in normal and water stress conditions. We found that the two key tonoplast aquaporins, TIP3;1 and TIP3;2, act antagonistically to modulate the response to ABA, with TIP3;1 being a positive and TIP3;2 a negative regulator. A third isoform, TIP4;1, which is normally expressed upon completion of germination, was found to play an earlier role during water stress. Seed TIPs also regulate the depth of primary dormancy and differences in the induction of secondary dormancy in a similar manner. We propose that the different responses of the seed TIP constitute a mechanism to influence germination under water stress and dormancy cycling in variable soil conditions.

## 1 INTRODUCTION

Water content in seeds changes dramatically during imbibition to drive radicle extension through its emergence from the seed coat to complete germination. This radicle growth during germination does not require cell division but is driven by gibberellic acid (GA)‐mediated expansion of cells in the hypocotyl (Stamm et al., 2017). Timing of germination completion is controlled by dormancy. During dormancy, cell expansion is blocked, and in nondormant seeds, this effect can be replicated by exogenous abscisic acid (ABA; Graham, & Graham, 2006; Penfield, Li, Gilday). Consequently, water uptake in seeds is influenced by the balance in sensitivity between ABA and GA. This ABA/GA balance regulates dormancy induction and relief resulting in shifting water potential thresholds for radicle emergence (Finch‐Savage & Leubner‐Metzger, 2006; Ni & Bradford, 1992). These thresholds change with dormancy status (Bradford, 2002).

The primary dormancy of seeds when dispersed from the parent plant is progressively lost in response to environmental signals, and seeds become sensitive to other signals (e.g., light) that allow germination to proceed. In the absence of these correct signals, seed dormancy is reinduced and seeds enter a state of secondary dormancy. In the field, water potential thresholds for radicle emergence change and follow the annual cycle of secondary dormancy driven by seasonal changes in the environment (Footitt, Douterelo‐Soler, Clay, & Finch‐Savage, 2011). During this cycle, as dormancy is reduced and seeds enter a shallow dormancy phase, water potential thresholds become more negative and seeds are sensitive to the environmental signals that remove the final layers of dormancy, enabling germination completion. In the absence of appropriate signals, seeds cycle back into the deep dormancy phase (water potential thresholds become less negative) of the dormancy continuum (Finch‐Savage & Footitt, 2017).

There has been speculation on a role for aquaporins in this process for a long time. For example, when aquaporin function is inhibited, the completion of seed germination is delayed (Vander Willigen, Postaire, Tournaire‐Roux, Boursiac, & Maurel, 2006). Regulation of water flow is also a regulatory component contributing to dormancy status that affects the progress of germination and its response to the environment, as well as its completion. The aquaporins present in seeds have been identified (Gattolin, Sorieul, & Frigerio, 2011; Vander Willigen et al., 2006), yet little is known about their function in the regulation of germination.

The three major higher plant aquaporin subfamilies are categorized based on localization and function; plasma membrane intrinsic proteins (PIPs), tonoplast intrinsic proteins (TIPs), and the nodulin 26‐like intrinsic proteins (NIPs; Maurel et al., 2015). TIPs consist of five subgroups that are present throughout higher plants, indicating they diverged early in higher plant evolution (Maurel et al., 2015). In *Arabidopsis*, these subgroups consist of 10 TIP isoforms that are developmentally and temporally expressed: three TIP1 (γ‐TIP), three TIP2 (δ‐TIP), the seed‐specific TIP3;1 and TIP3;2 (α‐ and β‐TIP, respectively), one TIP4 (TIP4;1, ε‐TIP), and one TIP5 (TIP5;1, ζ‐TIP; Johanson et al., 2001).

*TIP3;1* and *TIP3;2* (henceforth collectively referred to as *TIP3*) are highly expressed during seed maturation and early during germination. TIP3 are located on the tonoplast of seed protein storage vacuoles (PSV; Feeney, Kittelmann, Hawes, & Frigerio, 2018; Gattolin et al., 2011; Hunter, Craddock, Di Benedetto, Roberts, & Frigerio, 2007) and at the plasma membrane (Gattolin et al., 2011). In embryos from mature seeds, the protein expression patterns of TIP3;1 and TIP3;2 overlap (Gattolin et al., 2011). In contrast to TIP3, other aquaporins (including PIPs, TIPs, and NIPS) are expressed at low levels in dry seeds: Their levels are low at the end of seed maturation, with expression only increasing coincident with radicle emergence from the seed coat (Vander Willigen et al., 2006). It is therefore reasonable to hypothesize that in maturing and germinating seeds TIP3 may also be performing the role of PIPs by being present at the plasma membrane. It is not currently known how the dual localization of TIP3 occurs.

*TIP3* knockout/knockdown mutants have no obvious germination and growth phenotypes but do have a role in the maintenance of seed longevity (Mao & Sun, 2015). Whereas *Arabidopsis* embryos only appear to express the two TIP3 isoforms, another isoform, *TIP4;1*, is the first vegetative TIP expressed in the roots of germinated seedlings (Gattolin, Sorieul, & Frigerio, 2010; Gattolin, Sorieul, Hunter, Khonsari, & Frigerio, 2009). Transcriptionally, *TIP3* gene expression ceases before the completion of germination (i.e., radicle emergence from the seed coat), whereas *TIP4;1* expression begins post germination (Data S1; Dekkers et al., 2013).

ABA is intimately linked to the water relations of seeds with both ABA‐dependent and ‐independent signalling pathways reported during osmotic stress (Ni & Bradford, 1992). Regulation of these pathways involves phosphorylation/dephosphorylation cascades orchestrated by the SNF1‐related kinase 2 (SNRK2) family and protein phosphatase 2C (PP2C) family members that include ABA INSENSITIVE1 & 2 (ABI1 & 2; as reviewed in Yoshida, Mogami, & Yamaguchi‐Shinozaki, 2014; Hubbard, Nishimura, Hitomi, Getzoff, & Schroeder, 2010). Members of the SNRK2 family are differentially activated by phosphorylation in response to ABA, and osmotic and salt stress. Evidence for the involvement SNRK2s and phosphorylation in increasing water transport via aquaporins was seen in guard cells when PIP2;1 was phosphorylated by SnRK2.6, a component of ABA signalling (Grondin et al., 2015), and on phosphorylation of TIP3;1 in oocytes (Maurel, Kado, Guern, & Chrispeels, 1995). Although TIP3;1 was shown to facilitate water transport (Maurel et al., 1995), TIP3;2 was found to facilitate transport of the osmolyte glycerol rather than water (Li et al., 2008).

Dormancy has evolved to regulate germination under variable environmental conditions, and this process cannot be fully understood by experiments in controlled conditions alone. Therefore, in controlled laboratory experiments, we first explored the roles of TIP3;1, TIP3;2, and TIP4;1 by comparing sensitivity to ABA, base water potential for germination (i.e., the minimum water potential at which germination completion can occur), primary dormancy, and the induction of secondary dormancy in different knockout/knockdown and complemented lines. We then investigated their role in natural variable environments by extending our analysis to look at TIP3 gene expression and protein accumulation during dormancy cycling under field conditions and under a global warming scenario in a thermogradient tunnel. We discuss the roles of these aquaporins as seed dormancy changes and seeds germinate in response to the environment.

## 2 MATERIALS AND METHODS

### 2.1 Mutants, recombinant DNA, and transgenic plants

The T‐DNA lines *tip3;1* (SALK_053807.26.20), *tip3;2* (SALK_125353C), and *tip4;1* (SALK_050663) were obtained from the Nottingham Arabidopsis Stock Centre. The *tip3:1tip3:2* double mutant was produced by crossing homozygous single knockouts. Mutants were verified by PCR (see Table S1 for primer sequences). Complemented lines were produced by transforming *tip3;1tip3;2* with YFP‐TIP3;2 (Gattolin et al., 2011) and *tip4;1* with YFP‐TIP4;1 (Gattolin et al., 2009). Transgenic plants were selected on kanamycin plates.

### 2.2 Recombinant DNA

The construction of TIP3;2‐mCherry and TIP4;1‐YFP has been reported previously (Gattolin et al., 2009; Hunter et al., 2007). All constructs were introduced into Arabidopsis thaliana ecotype Col‐0 (N1092) by the floral dip method (Clough & Bent, 1998). Transgenic plants were either selected on kanamycin or directly identified by YFP fluorescence of seeds under a stereomicroscope.

### 2.3 Seed production of wild types, mutants, and complemented lines

The Arabidopsis mutants and transgenic lines used were in the Col‐0 (N1092) genetic background. All mutant and transgenic lines and the parental wild type (Col‐0) were produced at the same time. Seeds were sown directly onto growth medium (Levingtons F1 compost:silver sand:vermiculite 6:1:1) in P24 cellular trays (24 cells, each 5 × 5 × 5 cm). Trays sown with the lines and wild type were placed at 5°C in the dark for 3 days to reduce primary dormancy to the point at which dormancy can be completely removed by exposure to light. Trays were then transferred to a single growth cabinet at 22°/18°C, 16 hr L/8 hr D. As each plant bolted, the inflorescence was covered with a baguette bread bag. Seeds were harvested at maturity by hand threshing and cleaned seeds equilibrated for 5 days over a saturated Ca (NO_3_)_2_ solution that maintains a relative humidity of 55% RH at 20°C to produce an equilibrium moisture content of 6–10% on a dry weight basis. Seeds were then stored at −80°C until required.

Seeds of the Arabidopsis Cape Verde Island (Cvi) ecotype were produced in a heated glasshouse with supplemental lighting in 2007 and were harvested, processed, and then stored at −80°C as described elsewhere (Footitt et al., 2011).

### 2.4 Seed germination assays

Seeds were surface sterilized in 2.5% dilution of domestic bleach for 5 min and washed three times in water. Seeds were then placed (3 × 50 seeds) into boxes (124 × 88 × 22 mm; Stewart Plastics Ltd, UK) containing two sheets of Whatman 3MM chromatography paper (Camlab, UK) and 8 ml of water. Strips of nylon mesh (125‐μm mesh size, 45% open mesh; Plastok, UK) were then laid on the paper, and each replicate of seeds was placed on one of those individual strips. Boxes were then sealed inside freezer bags to minimize evaporation and wrapped in two layers of aluminium foil to exclude light and incubated at 5°C for 3 days to reduce primary dormancy to the point at which dormancy can be completely removed by exposure to light. The nylon strips holding the seeds were transferred to new boxes containing 25 ml of solution set at a range of water potentials (0 and −0.6 to −1.3 MPa) using PEG 8000. This PEG solution volume represents a solution volume/paper weight ratio greater than 3.55 and so minimizes the concentrating effect of filter paper on the solution (Hardegree & Emmerich, 1990). This liquid reservoir was accommodated beneath the seeds as follows. In the base of each box was placed a piece of glass‐drying mat (Nisbits Ltd, UK). The drying mat was an open lattice 3 mm deep to create space for the PEG solution. On top of this was placed nylon mesh (1‐mm mesh size; Plastok, UK) to support a single sheet of Whatman 3MM chromatography paper (Camlab, UK) that is then placed on top. The nylon strips bearing seeds were then laid on this paper. Boxes were then sealed inside freezer bags to minimize evaporation and incubated in the light at 15°C for up to 28 days to record germination. Radicle emergence was recorded as protrusion of the radicle through the seed coat and micropylar endosperm.

### 2.5 Confocal microscopy

Seeds were placed to germinate as above on water, −1.2‐MPa PEG, buffer (see ABA sensitivity experiments below), 175‐ or 250‐nM ABA in buffer in a growth cabinet set at 15°C and constant light. For observation, embryos were dissected from imbibed seeds before germination and at the completion of germination (once radicles had emerged from the seed coat). These embryos were mounted on a microscope slide in water and imaged with a Zeiss LSM 880 confocal microscope, using a 25× objective lens. YFP was excited at 514 nm and detected in the 525‐ to 585‐nm range. mCherry was excited at 561 nm and detected in the 565‐ to 640‐nm range. Simultaneous detection of YFP and mCherry was performed by combining these settings in the sequential scanning facility of the microscope, according to the manufacturer's instructions. Image processing and 3D reconstructions of *z*‐stacks, and tiles were performed with the Zeiss Zen (Blue edition).

### 2.6 Determining the base water potential for germination in TIP3 and TIP4;1 mutant and transgenic lines

The germination sensitivity of seeds to a range of water potentials at 15°C was determined as described above. The resulting data were used to determine the base water potential for germination completion for each line using hydrotime analysis (Bradford, 1990). Briefly, radicle emergence percentages were transformed to probits and plotted against the base or minimum water potential (Ψ_b_) permitting radicle emergence of percentage *g* (Ψ_b(g)_), where Ψ_b(g)_ = Ψ − (θ_*H*_/*t*_*g*_) enabling an estimate of Ψ_b(g)_ for the time to each percentage (*t*_*g*_) and by using different values of θ_*H*_ (hydrotime constant, MPa hr^−1^) to find the best fit. Radicle emergence percentage is based on viable seeds. From the resulting linear regression, the mean Ψ_b_ can be calculated (at probit [50% radicle emergence] = 0) and σ _Ψb_, the standard deviation of Ψ_b_, from the inverse of the slope (refer to Bradford, 1990, for full details).

### 2.7 ABA sensitivity experiments

Seeds were surface sterilized, and 3 × 40 seeds were placed on to nylon mesh strips in boxes containing water and two sheets of chromatography paper as above. They were then incubated at 5°C/dark for 3 days to reduce primary dormancy to the point at which dormancy can be completely removed by exposure to light. Nylon strips bearing seeds were then transferred to boxes containing 8 ml of 75‐nM (±) ABA (Sigma, UK) in 1.7‐mM citrate acid/3.3‐mM K_2_HPO_4_ buffer (pH 5.0) and incubated in the light at 25°C. This ABA concentration was selected as most effective in preliminary experiments using a range from 10 to 250 nM. ABA was dissolved in 100‐μl 0.1M KOH before preparing the stock solution in the buffer above. Germination was recorded as above for 7 days.

### 2.8 Thermodormancy and secondary dormancy induction in TIP gene mutants and transgenic lines

The Col‐0 wild type exhibits primary high temperature thermodormancy. To see if the mutants and complemented lines exhibited altered thermodormancy, seeds of all lines were surface sterilized, and 3 × 40 seeds were placed in boxes containing two sheets of chromatography paper and 8 ml of water as above and incubated at 15°C, 20°C, and 25°C in the light. Final germination was recorded as above after 14 days. Once primary dormancy has been removed (i.e., by cold treatment in these experiments), then secondary dormancy can be induced under conditions that inhibit germination completion. The role of the proteins encoded by the *TIP3;1*, *TIP3;2*, and *TIP4;1* genes in this secondary dormancy induction was also investigated. This used the laboratory dormancy induction protocol developed to facilitate the genetic dissection of dormancy induction and cycling in Arabidopsis (Footitt, Olcer‐Footitt, Hambidge, & Finch‐Savage, 2017) outlined below.

Induction of secondary dormancy in the wild type, Col‐0, the mutant and transgenic lines were tested as follows. Seeds were surface sterilized in the dark under a green safe light, and 3 × 40 seeds were placed on to nylon strips in boxes set up as used for investigating base water potential above and then cold conditioned at 5°C/−1.0 MPa/dark for 28 days to reduce primary dormancy to the point at which dormancy can be completely removed by exposure to light. Seeds were then transferred under a green safe light to fresh PEG‐8000 solution at −1.0 MPA and incubated at 25°C/dark for up to 14 days. At 0, 4, 7, 10, and 14 days at 25°C/dark, dormancy level was tested by recording germination following transfer of seeds to water at 25°C/light for up to 14 days. Dark germination was recorded at each transfer point. Full protocol details are given in Footitt et al. (2017). The time to induction of secondary dormancy in 50% (ID50) of the population was determined by plotting Log time (days) versus probit germination completion (%). The intercept of the regression line at 0 probit represents the ID50. Significant differences in ID50 between the Col‐0 wild type and each line were determined by analysis of variance.

### 2.9 TIP3;1 and TIP3;2 gene and protein expression analysis in the Cvi ecotype during dormancy cycling in the field

As seed dormancy cycling displays an annual rhythm in response to seasonal soil temperature patterns, we determined the transcriptional profile of the *TIP3;1* and *TIP3;2* genes and the level of TIP3 protein in seeds recovered over 12 months from field soil. Experiments using Cvi in the field were performed in 2007/2008 using the randomized block designs described previously (Footitt et al., 2011). Seeds were recovered from the soil in the morning of the day of harvest.

An additional experiment performed in 2012/13 used a thermogradient tunnel to investigate the impact of increased temperature during global warming on dormancy cycling. The use of this polyethylene tunnel (32 m long × 9 m wide) structure is described elsewhere (Footitt et al., 2017; Huang, Footitt, Tang, & Finch‐Savage, 2018; Wurr, Fellows, & Phelps, 1996). The basic operation involves monitoring the temperature outside the tunnel reacting to which an electronic climate control system operates fans generating opposing warmed and ambient air flows to establish and maintain an air temperature gradient from ambient at one end of the tunnel to *c*. ambient +4°C at the other end (Wurr et al., 1996). This represents a projected median emissions scenario for the local experimental area used in this work (West Midlands, UK) that indicates an increase in the summer mean temperature of 3.7°C by 2080 (UK Climate Change Projections, 2014). Air and soil temperatures were monitored continuously along the tunnel. Realistic seasonal and diurnal air and soil temperature fluctuations were therefore maintained within the tunnel but with varying degrees of simulated climate warming depending on the position along the tunnel.

Three independent biological replicates of Cvi seeds (50 mg each) from the same seed lot were used. These were placed in 5 × 5 cm nylon mesh bags and dispersed in 12‐g Ballotini balls (100‐ to 250‐μm diameter; Potters Ballotini Ltd, UK) as described in Footitt et al. (2011) and buried in October 2012 at 5‐cm depth in field soil in 17.5‐cm diameter square rigid black pots (Fargro, BHGS horticultural, UK). Eleven pots were placed at both the ambient end of the tunnel and the warm end, ambient +4°C. Seed samples were recovered from the pots and processed over a 12‐month period. Pots were watered at least once a week (more frequently in summer) to maintain soil moisture. Soil temperature was recorded at seed depth. For sample recovery, an individual pot from each end of the tunnel was transferred to a dark room and seeds recovered as described elsewhere (Footitt & Finch‐Savage, 2011). Samples were retained for protein analysis and stored at −80°C. Other samples from the same pots were used for dormancy testing at 5°C/light ±10 mM KNO_3_ (Footitt et al., 2011).

### 2.10 RNA Extraction and QPCR

QPCR of *TIP3;1* and *TIP3;2* gene expression was performed using the touchdown PCR thermal cycle: one cycle at 95°C for 10 min followed by 50 cycles at 95°C for 30s, 70°C (decreasing by 0.2°C/cycle to a target temperature of 67°C) for 30 s, and 72°C for 30 s. All other details regarding RNA extraction, QPCR, and analysis were described previously (Footitt, Muller, Kermode, & Finch‐Savage, 2015). Primer sequences for *TIP3* and reference genes are given in Table S2.

### 2.11 Western blot analysis of TIP3 expression in seeds during dormancy cycling

Total protein was extracted from frozen seeds (20 mg) in 300‐μl homogenization buffer (1‐mM EDTA, 0.2‐M NaCl, 0.1‐M TRIS‐HCl [pH 7.8], 2% *v*/v ß‐Mercapto ethanol, 0.2% *v*/v Triton X‐100, and a protease inhibitor tablet [Roche]) at 4°C. The homogenate was centrifuged at 15,000 g/10 min/4°C and the supernatant collected. Total protein was quantified using the Bradford Ultra (detergent compatible) protein assay kit (Expedeon, UK). SDS‐PAGE gels were run using 10‐μg total protein per lane with triplicate lanes for each sample. Protein was transferred to PVDF transfer membrane (Invitrogen) and probed with a custom TIP3 polyclonal antibody (GenScript, USA) based on the epitope CHQPLAPEDY as described (Jauh, Phillips, & Rogers, 1999). Positive bands were detected using ECL western blotting substrate (Promega) and quantified using an ImageQuant chemiluminescence detection system (GE Healthcare Life Sciences). Band intensity was normalized to the intensity of TIP3 detected in seeds prior to burial (time zero control sample).

### 2.12 Data analysis

One‐way analysis of variance was used to detect the differences between variates in response to ABA and thermodormancy. Time to 50% induction of dormancy was determined by regression analysis of probit transformed data followed one‐way analysis of variance. Linear regression analysis was used to identify correlations. Hydrotime analysis is described above and used probit transformed data with additional statistical analysis using paired *t* tests assuming unequal variance.

## 3 RESULTS

### 3.1 Effect of water stress and ABA on the timing of TIP3;2 and TIP4;1 protein accumulation

**Figure 1:**
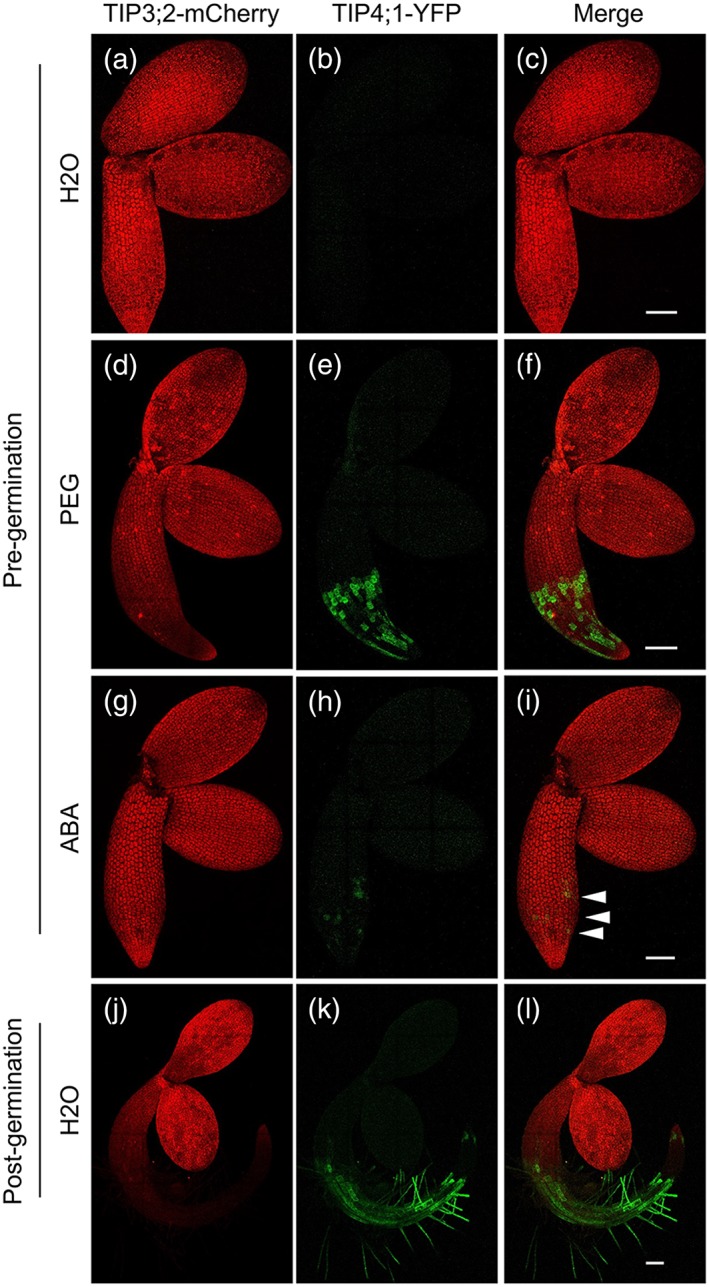
In the presence of osmotic stress and exogenous ABA, TIP4;1‐YFP accumulation is observed in radicle cells before germination. Seeds from a line coexpressing TIP3;2‐mCherry (red) and TIP4;1‐YFP (green) under their native promoters were imaged by confocal microscopy, after seed coat removal, both before (a–i) and after testa rupture allowing radicle emergence to complete germination (j–l). On water treatment, TIP4;1‐YFP was not observed before radicle emergence (a–c), but the signal appeared in the root elongation zone after its emergence (j–l). On −1.2‐MPa PEG (d–f) and 175‐nM ABA (g–i) treatments, the TIP4;1‐YFP signal was detectable in radicle cells before radicle emergence (arrowheads). TIP3;2‐mCherry is expressed throughout the embryo, but signal begins to decline after radicle emergence (a, d, g, j). Scale bar = 100 μm

To investigate the precise timing of TIP3;2 and TIP4;1 accumulation (after relieving seed dormancy by cold treatment), we germinated seeds of a transgenic line coexpressing TIP3;2‐mCherry and TIP4;1‐YFP under their native promoters on water at 20°C and constant light. Embryos or seedlings were imaged at different times before and after radicle emergence (Figure 1). Whereas TIP3;2‐mCherry accumulates throughout the embryo and labels PSV as they remodel to become lytic vacuoles during this time frame (Zheng & Staehelin, 2011; Figure 1a,d,g,j), TIP4;1‐YFP is not detectable in pre‐testa rupture embryos dissected from the seed coat (Figure 1b,c), but it appears in the epidermis and cortex of the root elongation zone soon after the radicle has emerged (Figures 1k,l and S1). When seeds were subjected to −1.2‐MPa PEG‐induced water stress (Figure 1d–f) or treated with 175‐ or 250‐nM ABA (Figure 1g–i), TIP4;1‐YFP became detectable in radicle cells in the root elongation zone of embryos dissected from seed coats before testa rupture and radicle emergence. The TIP4;1‐YFP signal was lower in seeds subjected to ABA. This demonstrates that TIP4;1 can be induced pre‐testa rupture by water stress or ABA treatment.

Given the unanticipated induction of TIP4;1 and its overlap with TIP3 prior to radicle emergence, we investigated the roles of all these isoforms in the germination response to water stress and ABA. We used a panel of T‐DNA insertion lines: *tip3;1*, *tip3;2*, *tip3;1tip3;2*, *tip4;1*. With the exception of *tip3;1*, where both gene and protein expression are down‐regulated by about 70% (Figure S2), all other lines were knockouts.

### 3.2 Contribution of TIP3 and TIP4;1 to germination under water stress

**Figure 2:**
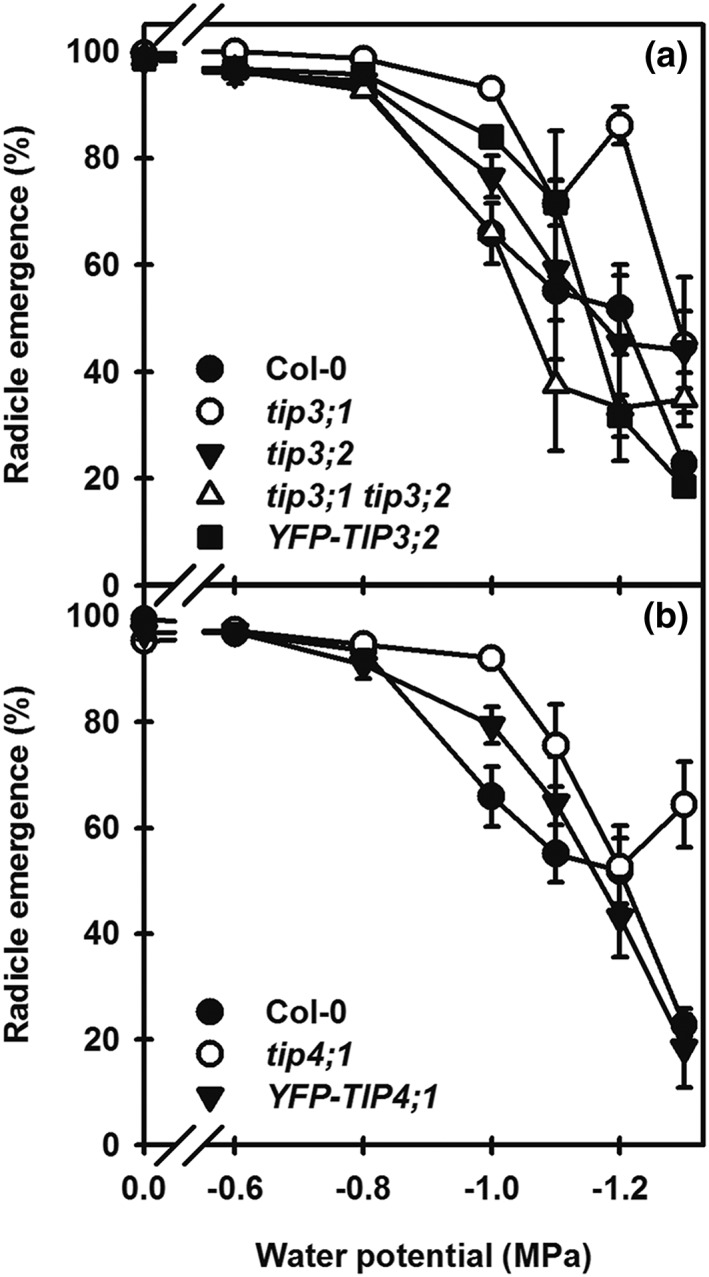
Response of TIP3 and TIP4;1 mutants and complemented lines to decreasing water potential. Seeds were incubated for 3 days at 5°C/dark on water to remove primary dormancy. Then seeds were placed to germinate for up to 28 days at 15°C/light on water potentials ranging from 0 to −1.3 MPa. (a) Response of tip3 mutant lines and the complemented line YFP‐TIP3;2. (b) Response of the tip4;1 mutant and the complemented line YFP‐TIP4;1. Each panel contains the Col‐0 wild‐type control. Data are mean ± SE (n = 3). Absence of error bars indicates SE is smaller than the symbol

To avoid the confounding influence of dormancy in these experiments, dormancy was first reduced by cold treatment so that exposure to light would remove the final layer of dormancy. The mutant lines were then imbibed in the light at water potentials (Ψ) ranging from 0 to −1.3 MPa. Germination completion (radicle emergence) of all lines declined with decreasing Ψ, but the decline was less marked in *tip3;1*, resulting in greater percentage radicle emergence at Ψs below −1.0 MPa (Figure 2a). When the *tip3;1 tip3;2* mutant was transformed with *YFP‐TIP3;2* under its native promoter (labelled *YFP‐TIP3;2*), the response was similar to the control (Col‐0) and the *tip3;2* mutant. In the case of *tip4;1*, radicle emergence declined less than in Col‐0 as Ψ decreased (Figure 2b). The complemented line *YFP‐TIP4;1* (*tip4;1* + *YFP‐TIP4;1*) responded the same as Col‐0.

To quantify the germination response of these nondormant mutant lines to Ψ, the data in Figure 2 were subjected to hydrotime analysis (Bradford, 1990) to determine the mean base water potential (Ψ_b_). This parameter summarizes the impact of reduced Ψ as the mean Ψ below, which germination is prevented in the population. The mean Ψ_b_ of Col‐0 (wild‐type control) was −1.272 MPa (Table 1). In *tip3;1,* consistent with its lower sensitivity to Ψ, its Ψ_b_ was lower (more negative) at −1.312 MPa, even though it is not a complete knockout, with some contribution from the residual *TIP3;1* expression expected. The Ψ_b_ of *tip3;2* was similar to Col‐0 whereas that of *tip3;1tip3;2* was higher and significantly different (*P* < .05) from Col‐0 and *tip3;1* (no other significant differences were found). For the *tip3;1 tip3;2* double mutant complemented with *YFP‐TIP3;2* (*YFP‐TIP3;2*), Ψ_b_ was similar to Col‐0. Overall, in comparison with Col‐0, Ψ_b_ was reduced in *tip3;1* and increased in lines carrying *tip3;2*. Other lines had similar Ψ_b_ to the Col‐0. This indicates that the phenotype of the double mutant is nonadditive. Therefore, we conclude that rather than acting redundantly, both TIP3 isoforms are required for normal osmotic regulation in seeds. In the case of *TIP4;1,* the mutant line *tip4;1* had a Ψ_b_ of −1.326 MPa, therefore like *tip3;1* was more resistant to low water potential, indicating a role for *TIP4;1* in the seeds' response to their osmotic environment. However, this difference was not significant (*P* < .05).

**Table 1:** Hydrotime analysis of the germination response to osmotic stress of mutant and complemented TIP3 and TIP4;1 lines

Knockout/down and complemented lines	Mean base water potential (Ψ_b_; MPa)	Standard deviation (σΨ_b_)
Col‐0 (wild type)	−1.272^*^	0.222
*tıp3;1*	−1.312^**^	0.199
*tıp3;2*	−1.254	0.262
*tıp3;1 tıp3;2*	−1.175^*, **^	0.200
*YFP‐TIP3;2*	−1.260	0.171
*tıp4;1*	−1.326	0.228
*YFP‐TIP4;1*	−1.253	0.205

*Note*. Table shows the mean base water potential (Ψ_b_) of the wild type (Col‐0) and all mutant and complemented lines. Ψ_b_ is the minimum water potential at which germination completion can occur. As Col‐0 is the parental line of all mutants and complemented lines, the Col‐0 hydrotime constant (θ_*H*_) 34.352 MPa hr^−1^ was used as a standard for all lines in the analysis. Values followed by the same ^*^ or ^**^ are significantly different (*P* < .05) from one another determined by comparisons between *tip3* lines and Col‐0, and *tip4* lines and Col‐0 using paired *t* tests assuming unequal variance.

### 3.3 Response of TIP3 and TIP4;1 to ABA

**Figure 3:**
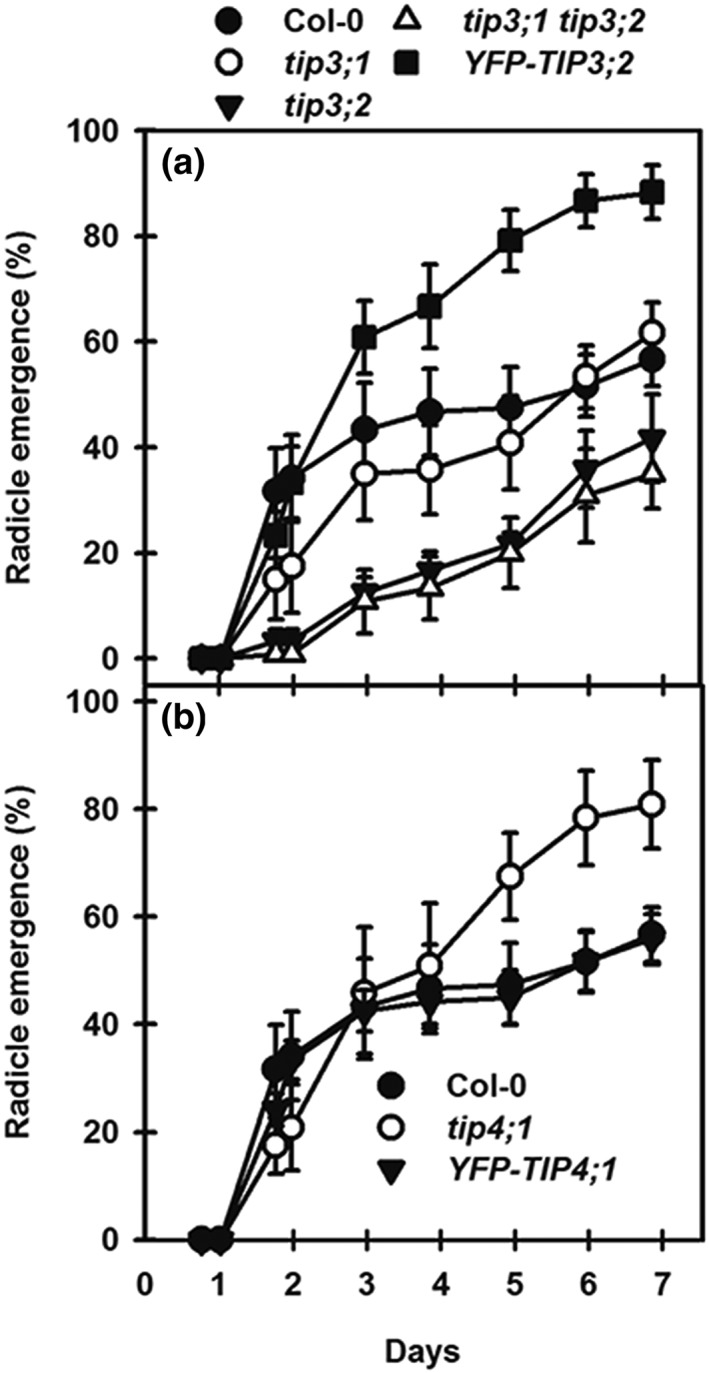
Cumulative radicle emergence of the TIP3 and TIP4;1 mutants and complemented lines in the presence of 75‐nM ABA. Seeds were incubated for 3 days at 5°C/dark on water to remove primary dormancy then transferred to 75‐nM ABA in buffer at pH 5.0 and cumulative radicle emergence recorded during incubation at 25°C/light over 11 days. (a) Response of the TIP3 single and double mutants and the complemented line YFP‐TIP3;2. (b) Response of the tip4;1 mutant and the complemented line YFP‐TIP4;1. In both cases, the wild type is Col‐0. Data are mean ± SE (n = 3). Absence of error bars indicates SE is smaller than the symbol

The results above point to nonredundant functions for TIP3;1 and TIP3;2 and a role for TIP4;1 during germination under water stress. As ABA is integral to the response to osmotic stress, we tested the germination response of nondormant TIP mutants to ABA (Figure 3). Therefore, dormancy was first reduced by cold treatment as above, making seeds light‐sensitive only. Seeds in buffer controls germinated to greater than 95% by 48 hr on transfer to 25°C in the light. The germination responses to ABA of Col‐0, *tip3;1*, and *tip3;2* were not significantly different (*P* < .05). However, the reduced germination of *tip3;2* indicated it was ABA hypersensitive. The *tip3;1tip3;2* line was ABA hypersensitive and significantly different from *tip3;1* (*P* < .05; Figure 3a). This indicates that in the absence of TIP3;2, the plant is more sensitive to ABA, indicating TIP3;1, as the sole TIP3, is a positive regulator of ABA responses. In contrast, the *tip3;1tip3;2* line complemented with *YFP‐TIP3;2* was significantly (*P* < .05) ABA hyposensitive in comparison with Col‐0 and other *TIP3* lines. This indicates that the isoform balance is shifted towards TIP3;2 leading to ABA hyposensitivity and TIP3;2 acting as a negative regulator of ABA responses. Here, TIP3;1 and TIP3;2 therefore act antagonistically in the response to ABA.

The *tip4;1* mutant had increased radicle emergence in the presence of ABA (but not significantly, *P* < .05), indicating ABA hyposensitivity (Figure 3b). As *TIP4;1* is induced pregermination by ABA (Figure 1), it appears to play a role in stress regulation prior to germination completion.

### 3.4 Genes coexpressed with TIP3 isoforms in the endosperm and radicle of germinating Arabidopsis seeds

To evaluate how gene expression of *TIP3;1 and TIP3;2* is coordinated in the seed, we interrogated publicly available microarray data. Both genes are expressed in the endosperm and radicle with expression strongly correlated (*R* > .932) with a number of other genes (Table 2). More genes correlate with *TIP3;2* than *TIP3;1*, with a small number correlating with these isoforms in both tissues. When these coexpressed genes are characterized regarding response to dormancy/germination and ABA/GA, the majority are up‐regulated during dormancy and/or in response to ABA (Table 3). In contrast *TIP4;1* is only expressed in the radicle and is coexpressed with only 59 genes; none of which are up‐regulated during dormancy or by ABA. Identities of the *TIP3* and *TIP4* coexpressed genes are given in Data S2.

**Table 2:** Genes coexpressed with TIP3 isoforms in the endosperm and radicle of germinating Arabidopsis seeds

Gene	Endosperm only	Radicle only	Endosperm and radicle
*TIP3;1*	296	275	66
*TIP3;2*	347	550	145

*Note*. Data represent the number of genes coexpressing with *TIP3;1* or *TIP3;2* if their correlation of expression is greater than .932. Data show the number of genes coexpressing with *TIP3* isoforms only in the endosperm and radicle respectively and those coexpressed with them in both tissues. Data were extracted from endnet (http://netvis.ico2s.org/dev/endonet/#/) and radnet (http://netvis.ico2s.org/dev/radnet/#/) accessed from http://ssbvseed01.nottingham.ac.uk/efp_browser/efpWeb.cgi (Dekkers et al., 2013).

**Table 3:** Classification of genes coexpressed with TIP3 isoforms in the endosperm and radicle of germinating Arabidopsis seeds in Table 2

Classification	TIP3;1		TIP3;2	
	Endosperm	Radicle	Endosperm	Radicle
Dormant up	116	131	219	260
Germination up	8	3	6	5
ABA down	2	2	3	13
ABA up	111	77	145	86
GA down	25	34	34	49
GA up	0	1	1	0

*Note*. Data represent genes coexpressing with *TIP3;1* or *TIP3;2* if their correlation of expression is greater than .932. Data were extracted from endnet (http://netvis.ico2s.org/dev/endonet/#/) and radnet (http://netvis.ico2s.org/dev/radnet/#/) accessed from http://ssbvseed01.nottingham.ac.uk/efp_browser/efpWeb.cgi (Dekkers et al., 2013). See Data S2 for details of coexpressed genes.

#### 3.4.1 Contribution of TIP3 and TIP4;1 to primary dormancy

**Figure 4:**
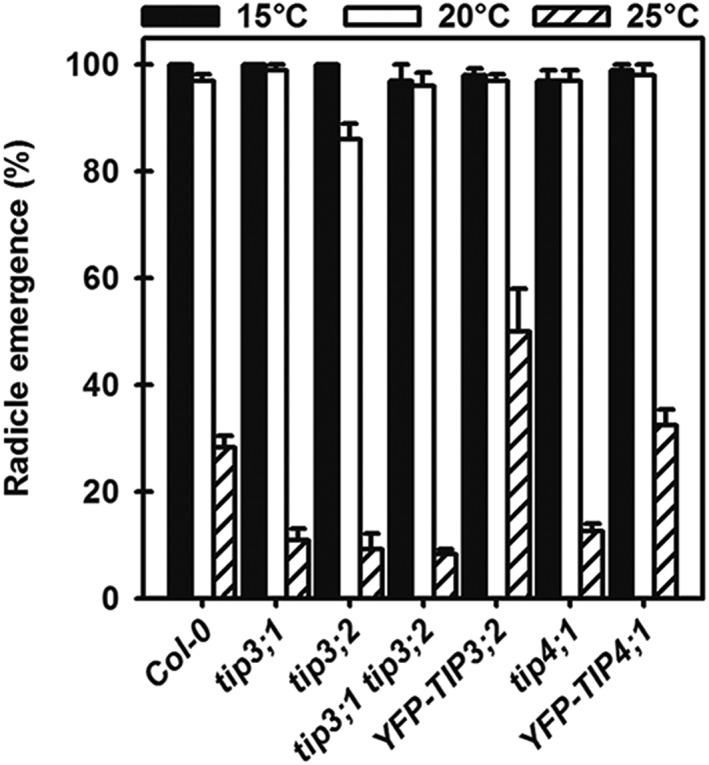
Thermodormancy in the TIP3 and TIP4;1 mutants and complemented lines. Seeds of all lines and the Col‐0 wild‐type control were incubated on water at 15°C, 20°C, and 25°C in the light. Final numbers of seeds with radicle emergence were recorded after 14 days. Data are mean ± SE (n = 3)

In the experiments above, primary dormancy (i.e., dormancy present at seed shedding) was removed to avoid its confounding effect on germination completion. As the *tip3* and *tip4;1* mutants show differing ABA responses to wild type and complemented lines in nondormant (cold treated) seeds (Figure 3), we tested the primary thermodormancy (temperature‐related dormancy level) in untreated seeds. At 15°C and 20°C, all lines germinated in excess of 96%, except for *tip3;2* whose germination declined significantly (*P* < .05) to 86% at 20°C (Figure 4). This shows thermo‐dormancy was present at a lower temperature in this mutant. At 25°C, the mutant lines (single and double) all exhibited the same level of thermodormancy, which was significantly (*P* < .05) deeper than in the complemented lines and Col‐0 (Figure 4). The complemented line *YFP‐TIP4;1* was not significantly different from Col‐0, whereas in the complemented line YFP‐TIP3;2, radicle emergence was significantly higher (*P* < .05) than in Col‐0. This indicates that all three TIPs tested have roles in primary dormancy, because their removal or down‐regulation enhanced dormancy.

#### 3.4.2 Contribution of TIP3 and TIP4;1 to secondary dormancy

**Figure 5:**
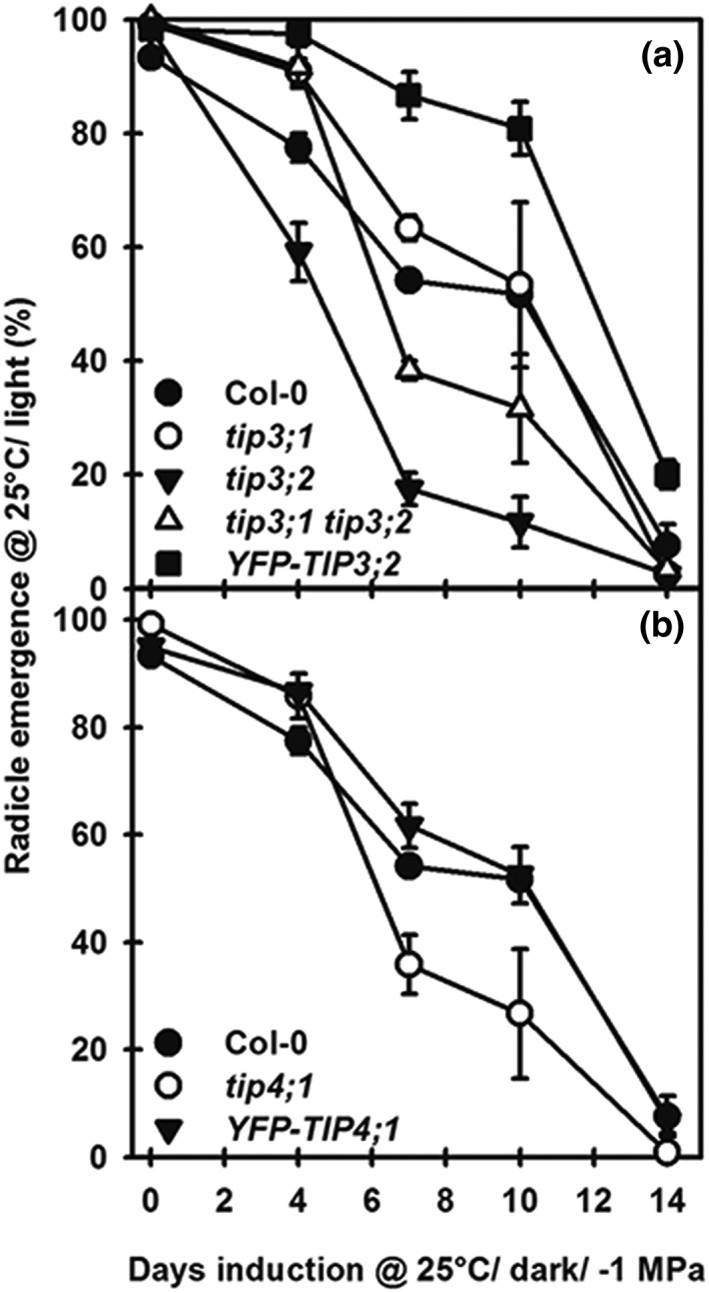
Induction of secondary dormancy in the TIP mutants, complemented lines, and the Col‐0 wild‐type control. Following 5°C/dark at −1.0 MPa for 28 days, seeds were transferred to −1.0 MPa and incubated in the dark at 25°C for 14 days. At increasing intervals, dormancy status was determined by transferring seeds to water at 25°C/light and recording radicle emergence for 14 days. (a) TIP3 mutants and the complemented line YFP‐TIP3;2. (b) TIP4;1 mutant and complemented line YFP‐TIP4;1. Seed data are mean ± SE (n = 3). Absence of error bars indicates SE is smaller than the symbol

The response of aquaporin mutants and complemented lines was further investigated during the induction of secondary dormancy. For these experiments, primary dormancy was first reduced by incubating seeds at 5°C/−1 MPa/dark for 28 days. At the end of this cold conditioning phase, there was no radicle emergence in the dark. However, radicle emergence was greater than 93% upon transfer to water at 25°C/light, showing dormancy was fully removed on exposure to light. At the end of cold conditioning, seeds were transferred (in the dark under a green safe light) to a fresh PEG‐8000 solution at −1 MPa to maintain the same water potential and incubated for up to a further 14 days at 25°C/dark. Under these conditions (−1 MPa/25°C/dark) germination on transfer to the light progressively declines as secondary dormancy is induced (Figure 5). The mutant *tip3;2* was the most sensitive to secondary dormancy induction and the complemented *YFP‐TIP3;2* line the most resistant. The other lines responded similarly to Col‐0. To summarize these results, the rate of induction (time to 50% induction of secondary dormancy [ID50]) was calculated (Table 4). ID50 of *YFP‐TIP3;2* was significantly slower (*P* < .01) than all other lines, whereas *tip3;2* was significantly faster than all other lines (*P* < .01). In the case of TIP4;1, *tip4;1* was more sensitive to secondary dormancy induction than Col‐0 but not significantly (*P* < .05). However, it was significantly different from *YFP‐TIP4;1* (*P* < .01; Figure 5 and Table 4).

**Table 4:** Time to 50% induction of secondary dormancy (ID50) in the mutant and complemented TIP3 and TIP4;1 lines

Knockout/down and complemented lines	Mean time to 50% induction of secondary dormancy (ID50; day)
Col‐0 (wild type)	7.948 ± 0.380 a,c
*tip3;1*	8.521 ± 0.371 a
*tip3;2*	4.977 ± 0.051
*tip3;1 tip3;2*	7.200 ± 0.445 c
*YFP‐TIP3;2*	11.668 ± 0.035
*tip4;1*	6.825 ± 0.197 c
*YFP‐TIP4;1*	8.709 ± 0.351 a

*Note*. Table shows the mean ID50 in response to water stress (−1 MPa) at 25°C in the dark of the wild type (Col‐0) and all mutant and complemented lines. Data are the mean ± standard error (*n* = 3). All values are significantly different at *P* < .05, except when followed by the same letters.

In summary, TIP3;1 and TIP3;2 contributed antagonistically to the induction of secondary dormancy, arguably resulting from their opposing responses to ABA, which drives secondary dormancy induction. As in primary dormancy, TIP3;2 has a large negative effect on dormancy induction, consistent with a reduced response to increasing ABA (Figures 3 and 5), whereas TIP3;1 promotes secondary dormancy induction. The *TIP4;1* mutant increased dormancy induction but not significantly. These results reveal a role for aquaporins in induction and relief of dormancy.

#### 3.4.3 TIP3 gene expression under natural variable soil conditions during dormancy cycling

**Figure 6:**
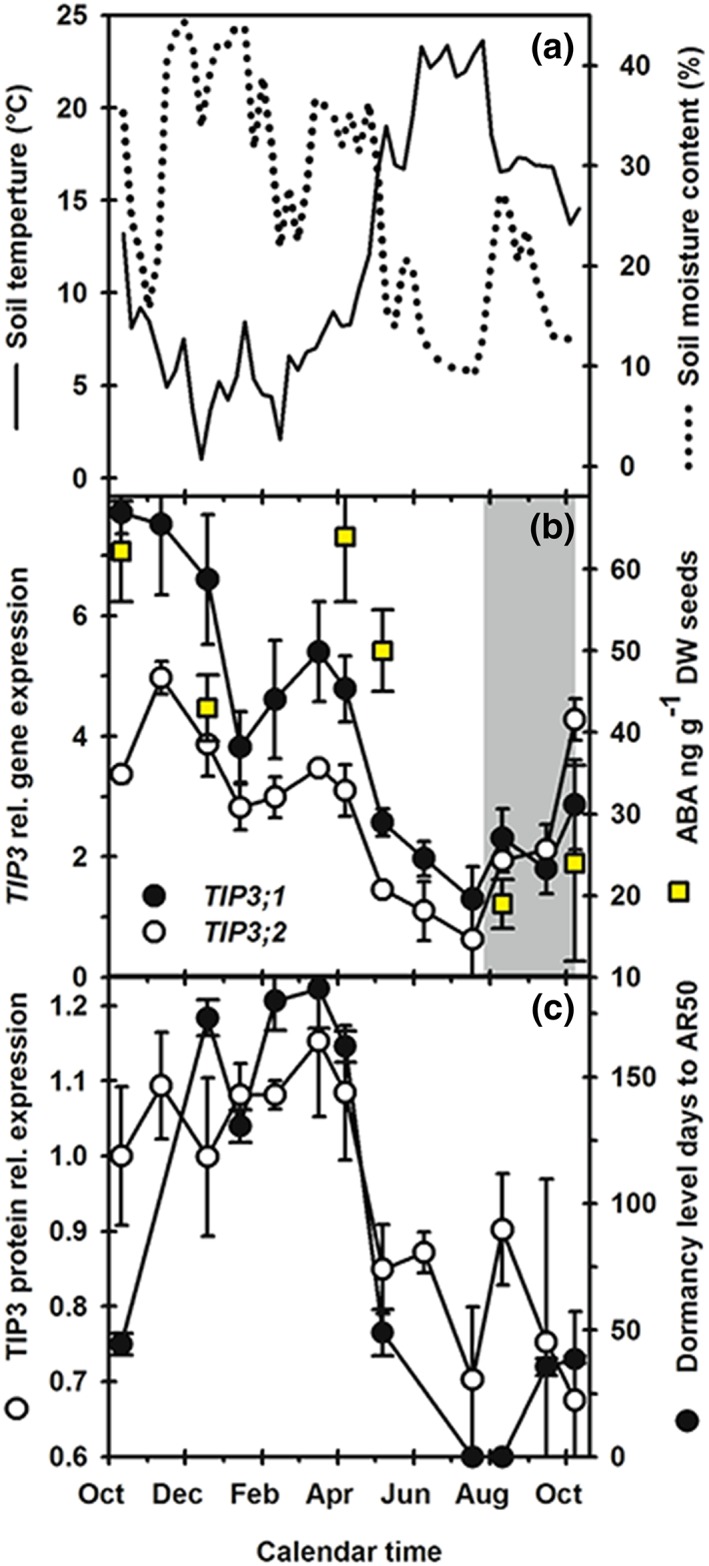
Seasonal patterns of TIP3;1 and TIP3;2 gene expression in seeds of the Arabidopsis ecotype Cvi buried in field soils. (a) Soil temperature and moisture content at seed depth. (b) Transcription profiles of TIP3;1 and TIP3;2 relative to the control, and ABA content of seeds prior to burial and at 5 points during the annual dormancy cycle in the field. There was regular soil disturbance to expose seeds to light, and the grey‐shaded area represents the period when this stimulated seedling emergence in the field. (c) TIP3 protein expression relative to that at burial and depth of dormancy ([AR50] days of dry after‐ripening [20°C/dark] to give 50% germination at 20°C in the light) in recovered Cvi seeds. Data in (a), and ABA levels and depth of dormancy are from Footitt et al. (2011). Seed data are mean ± SE (n = 3 biologically independent replicates). Absence of error bars indicates SE is smaller than the symbol [Colour figure can be viewed at wileyonlinelibrary.com]

As laboratory‐based tests showed a role for TIP3 isoforms in dormancy regulation, we examined their response in microarray data where dormancy was set at different levels in the laboratory (Cadman, Toorop, Hilhorst, & Finch‐Savage, 2006; Finch‐Savage, Cadman, Toorop, Lynn, & Hilhorst, 2007). These data showed that of the aquaporins, only *TIP3;1* and *TIP3;2* had consistently high transcript levels as dormancy levels changed (Data S3). We therefore looked at changes in *TIP3* transcript profiles during seasonal dormancy cycling in the soil. Soil temperature and moisture content was recorded at seed depth in the field (Figure 6a). We measured TIP3 gene and protein expression patterns in buried seeds of the deeply dormant *Arabidopsis* ecotype Cvi. On adjacent plots, soil was disturbed regularly to expose seeds to light and seedling emergence recorded. In seeds recovered from undisturbed plots, *TIP3;1* and *TIP3;2* had similar transcript profiles over the annual cycle (Figure 6). *TIP3;1* expression was greater than *TIP3;2* except when seedling emergence was observed in disturbed plots (Figure 6b, shaded area; Footitt et al., 2011). Using an antibody that recognizes both TIP3 isoforms (Jauh et al., 1999), we found that the level of TIP3 protein followed a similar profile (Figure 6C); both gene expression and protein accumulation were high when soil temperature was low and dormancy levels were high (Figure 6). Then expression of both decreased when dormancy decreased with rising soil temperature before increasing with dormancy as soil temperature decreased late in the year. Expression of the TIP3 genes and protein correlated negatively and significantly with soil temperature (*P* < .01 or greater; Table S3), but only *TIP3;1* gene expression and TIP3 protein had positive and significant correlations with dormancy levels (AR50; *P* < .001 and *P* < .01) and each other (*P* < .01). The pattern of gene and protein expression was also consistent with ABA level trends (Figure 6b,c). Both isoform transcripts responded to soil moisture, but only protein levels had a significant positive correlation (*P* < .001; Table S3). These results indicate that under “natural” variable conditions in the soil, TIP3 respond to environmental signals known to modulate dormancy via alteration of both ABA levels and sensitivity (Finch‐Savage & Footitt, 2017).

#### 3.4.4 TIP3 protein accumulation during dormancy cycling at different temperatures in a global warming scenario

**Figure 7:**
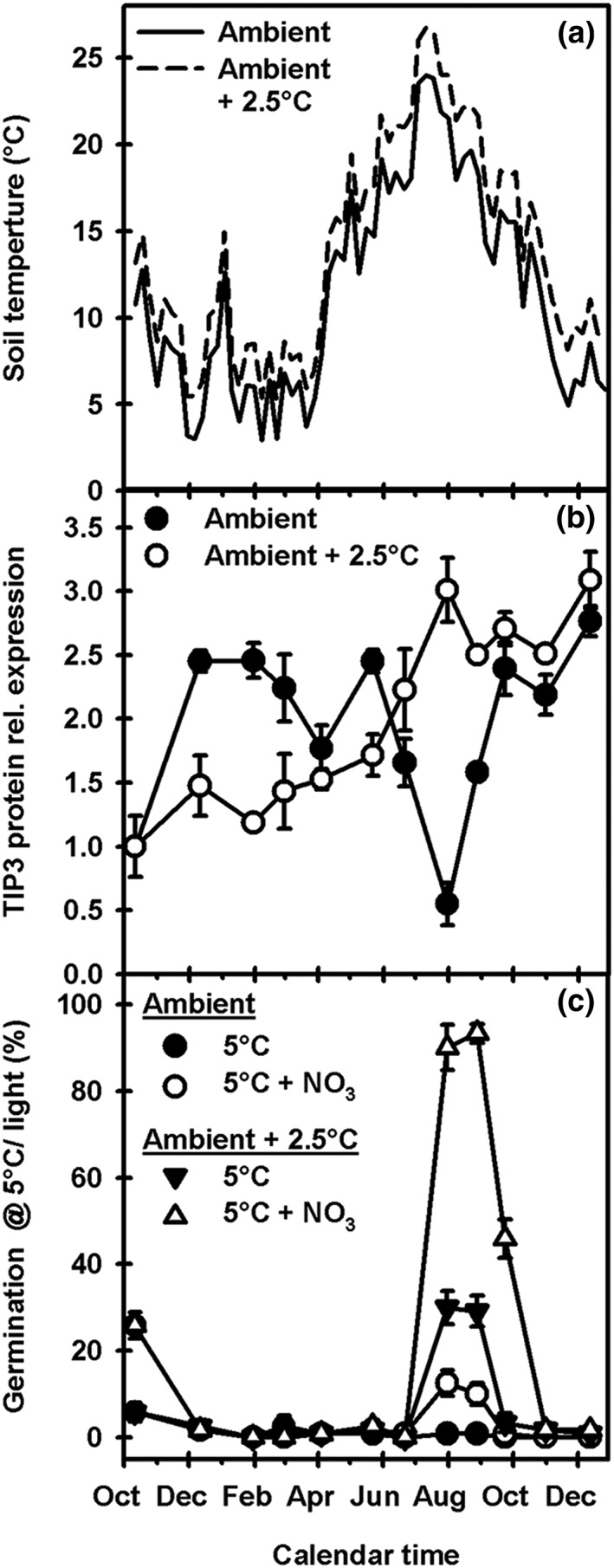
Impact of global warming on the seasonal patterns of TIP3 protein expression and dormancy in buried seeds of the Arabidopsis ecotype Cvi. Seeds were buried in pots at opposite ends of a thermogradient tunnel so they experienced ambient temperature conditions or ambient +2.5°C the projected temperature for 2080. (a) Soil temperatures at seed depth. (b) TIP3 protein profiles in seeds undergoing dormancy cycling under both temperature regimes. (c) Percentage radicle emergence from recovered seeds on water or 10‐mM KNO_3_ at 5°C in the light. Seed data are mean ± SE (n = 3). Absence of error bars indicates SE is smaller than the symbol

In the field, TIP3 gene and protein expression levels in seeds responded predominantly to temperature but also to soil moisture (Figure 6). As temperature is the primary environmental signal driving dormancy cycling, we looked at how TIP3 protein levels are affected by temperature changes in a global warming scenario. Experiments using Cvi seeds were performed in a thermogradient tunnel under a scenario representing projected increases in air temperature between the present day (ambient) and year 2080 (ambient +4°C). This scenario produced a mean difference in annual soil temperature at seed depth along the tunnel of 2.5 ± 0.1°C (Figure 7a). Plots were irrigated to remove the influence of variable soil water content. Accumulation of TIP3 protein under ambient soil conditions followed a cycling pattern that significantly negatively correlated (*P* < .05) to the annual temperature cycle, to produce the lowest protein level in August (Figure 7b). This is similar to the *TIP3;1* and *TIP3;2* transcript and TIP3 protein profiles seen in the field experiment (Figure 6). In contrast, at the warm end of the tunnel representing predicted 2080 temperatures (soil temperature is ambient +2.5°C), TIP3 levels increased to a peak in August. This is reflected in the differences in peak radicle emergence seen in recovered seeds placed to germinate at 5°C with and without nitrate (Figure 7c). Nitrate is an environmental signal that reduces dormancy and enhances the ability of light to remove the final layer of dormancy (Finch‐Savage & Footitt, 2017). Under ambient conditions, seeds had low sensitivity to nitrate and low radicle emergence, whereas ambient +2.5°C soil temperatures had a dramatic impact resulting in higher sensitivity to nitrate and consequently a greater increase in radicle emergence (Figure 7c). The predicted increase in soil temperature significantly reduced dormancy (*P* < .01; germination on water at 5°C in the light) and significantly increased sensitivity to the environmental signal nitrate (*P* < .01). This change in dormancy was reflected in a significant (*P* < .01) change in TIP3 protein levels at this point (July 31). As other environmental variables did not differ, changes in TIP3 protein levels were driven by temperature.

## 4 DISCUSSION

Here, we investigated the physiological roles of TIP3;1, TIP3;2, and TIP4;1 aquaporins in seeds exposed, in controlled conditions, to environmental signals and stresses (water and temperature) they would experience in their natural environment. We then observed TIP3 gene expression and protein levels during annual dormancy cycles as seeds respond to the variable soil environment in the open field, and in the different temperature scenarios of a simulated global warming experiment. Our key findings are (a) under control conditions, TIP3;1 and TIP3;2 are the only aquaporins present in the embryo prior to germination completion and are then followed by expression of TIP4;1; (b) TIP3 act antagonistically to modulate the response to ABA; TIP3;1 is a positive responder, and TIP3;2 is a negative responder; (c) this sensitivity is reflected under water stress by *tip3;1* tending to have greater resistance and the most negative base water potential; (d) the depth of primary dormancy and differences in the induction of secondary dormancy of the *tip3* mutants are consistent with those seen on exposure to ABA; (e) in the absence of water stress, TIP4;1 accumulation is only seen post germination but, under water stress and in the presence of exogenous ABA, TIP4;1 is observed prior to testa rupture and germination completion. This explains why the *tip4;1* mutation has a dormancy and germination phenotype when subjected to water and temperature stress and to ABA; (f) under water stress, TIP4;1 plays a role in stress regulation prior to germination completion but acts differently from TIP3;1 to similarly affect resistance to reduced Ψ.

Thus, widespread expression of TIP3 during germination was observed, yet knockouts of the TIP3;1 and TIP3;2 genes were viable and indeed had little impact on germination under control nonstressed conditions. This indicates that the principal function of these seed‐specific proteins is most likely manifesting during maturation, dehydration, and subsequent rehydration, when seeds are subject to significant changes to water status and stress. Nevertheless, a range of significant phenotypes is summarized above when seeds are subjected to stresses that can occur following shedding in the soil. Below, we discuss how these TIPs may exert influence on germination timing via differing contributions to the sensitivity of dormancy induction and relief in response to environmental stress.

### 4.1 TIP3 isoforms exhibit contrasting responses when exposed to ABA and osmotic stress

The TIP3 isoforms are only transcriptionally expressed in the embryo and endosperm prior to the completion of germination, although the protein persists for several days post germination (Dekkers et al., 2013; Gattolin et al., 2011). As such, it is possible they play important roles in the water relations of seeds upon entry into the soil environment. Increased ABA levels and sensitivity to ABA are integral to dormancy, germination completion, and overall plant responses to osmotic stress (Ni & Bradford, 1992; Schopfer & Plachy, 1984; Yoshida et al., 2015). However, in seeds, ABA has an independent synergistic effect with reduced Ψ (Finch‐Savage & Leubner‐Metzger, 2006; Ni & Bradford, 1992). Thus, ABA dependent changes in dormancy and germination result from shifting water potential thresholds for radicle emergence (Ni & Bradford, 1992). Additionally, seed sensitivity to ABA has been shown to increase with temperature (Gonai et al., 2004). Here, when primary dormant seeds were incubated at increasing temperatures, thermodormancy increased more at 25°C in the mutant lines than in the wild type. When primary dormancy was relieved, the resulting “nondormant” seeds showed differing sensitivities to exogenous ABA between lines when incubated at 25°C, consistent with an increasing sensitivity to ABA at this temperature. In the control (minus ABA), germination is complete in all lines, but in the presence of ABA, *tip3;2* and *tip3;1 tip3;2* (i.e., only TIP3;1 is present) were ABA hypersensitive. This demonstrates that TIP3;1 responds positively to increasing sensitivity to ABA thereby reducing germination. Even the residual levels of TIP3;1 from the leaky *tip3;1* line in *tip3;1 tip3;2* (about 30% of wild type, Mao & Sun, 2015; Figure S1) are sufficient to repress germination to the levels seen in *tip3;2*. In contrast, ABA hyposensitivity was seen when *tip3;1 tip3;2* was complemented with *YFP‐TIP3;2*. This indicates that TIP3;2 acts in either a neutral or negative fashion to the seeds increased sensitivity to ABA. Therefore, the TIP3 isoforms have an antagonistic relationship during changing sensitivity to ABA. As activation of *TIP3;1* and *TIP3;2* expression is ABI3 (ABA INSENSITIVE3) dependent in the presence of ABA (Mao & Sun, 2015), this provides an efficient mechanism for regulating water transport in response to environmentally driven changes in sensitivity to ABA.

### 4.2 TIP4;1 response to ABA, osmotic stress, and dormancy induction

Previously, the *TIP4;1* transcript was shown to be expressed in the radicle post germination (Dekkers et al., 2013) and was found to be expressed in the epidermal and cortical cells at the base of the elongation zone in roots from 8‐day‐old seedlings (Gattolin et al., 2009). We confirm here that, in the absence of stress, TIP4;1‐YFP is only detectable after radicle emergence. However, in the presence of ABA or osmotic stress, TIP4;1‐YFP was seen prior to radicle emergence (Figure 1). This response may result from an osmotic priming effect, which can advance aspects of germination when radicle emergence and germination completion is prevented. However, in this case, it is interesting that an event not normally occurring until the postgermination phase has been advanced to have some influence on the germination process. Germination of *tip4;1* was hyposensitive to ABA and osmotic stress, with the complemented line exhibiting wild‐type behaviour. Although induction of secondary dormancy in *tip4;1* is more rapid than in the wild type or complemented line the difference is not significant, suggesting it is secondary to the impact of TIP3. This is supported by the basal level of *TIP4;1* transcripts seen in microarrays of dormancy cycling (Data S3; Cadman et al., 2006; Finch‐Savage et al., 2007).

### 4.3 The role of TIP3 in dormancy regulation

Changing dormancy levels in seeds are predominantly induced by temperature but are also influenced by water stress. Response to these environmental signals is orchestrated by the balance between the ABA and GA signalling pathways.(Finch‐Savage & Footitt, 2017; Finch‐Savage & Leubner‐Metzger, 2006; Footitt et al., 2017; Footitt & Finch‐Savage, 2011). Publicly available microarray data show that based on coexpression with other genes, the TIP3 isoforms sit on the ABA side of the ABA/GA balance. We show the TIP3 isoforms are involved in the seed's responses to ABA and water stress and have an impact on thermodormancy in primary dormant seeds and in particular the induction of secondary dormancy.

The results presented here indicate a role for aquaporins in regulation of the dormancy continuum. For example, these findings suggest the regulation of *TIP3* expression and function is responsive respectively to ABA and sensitivity to ABA (ABA signalling); both of which increase in the deep dormancy phase and decline in the shallow dormancy phase of the dormancy cycle (Finch‐Savage & Footitt, 2017). This is supported by the ABA‐induced expression of the Oryza sativa *TIP3* genes and the repression of *OsTIP3;1* by ABI4 (Han et al., 2018). During dormancy cycling in the field *ABI4* expression peaks in the summer months (shallow dormancy phase; Footitt et al., 2011) and is significantly negatively correlated with the *TIP3* genes and protein (*P* < .01 for TIP3;1 and *P* < .001 for the TIP3 protein; Table S3). ABI4 also represses lipid mobilization in the embryo and is induced via the ABA‐mediated sugar signalling pathway, which involves DOG1 (Penfield et al., 2006; Teng, Rognoni, Bentsink, & Smeekens, 2008). Additionally, in germinating Hordeum vulgare seeds, ABA‐induced *HvTIP3;1* expression delays fusion of the PSVs during the vacuolation process in aleurone cells (Lee et al., 2015). As vacuoles are essential in the generation of turgor to drive radicle emergence (Obroucheva, Sinkevich, Lityagina, & Novikova, 2017), *TIP3;1* can be seen as a negative controller in the initiation of the germination process until dormancy is removed. This illustrates the highly sophisticated integration of dormancy regulation with the environment seen in the dormancy cycling experiments reported here.

### 4.4 TIPs and the timing of dormancy and seed germination in response to the environment

Our knowledge of the precise functioning of TIPs is currently limited. Li et al. (2008) investigated the permeability to water and glycerol of a rice TIP representative from each of five different groups (TIP1–5) in a *Xenopus* oocyte system. They found OsTIP3;2 had glycerol transport activity only, whereas OsTIP4;1 had dual functions in both glycerol and water transport. In another oocyte study, OsTIP3;1 water transport activity was found to be no different from the negative control and so did not transport water (Hayashi, Ishikawa‐Sakurai, Murai‐Hatano, Ahamed, & Uemura, 2015). In contrast, Maurel et al. (1995) showed that AtTIP3;1 (αTIP) did facilitate water transport. It is possible that both can occur as Utsugi, Shibasaka, Maekawa, and Katsuhara (2015) showed in oocyctes that HvTIP3;1 singly was found not to have water transport activity but did when coexpressed with HvTIP1;2 (Utsugi et al., 2015). Thus, TIPS can coregulate water and solute transport, but the precise function of individual TIPs may differ and be influenced by the presence of other TIPS.

On the basis of current understanding, we propose that TIP3 isoforms can act in concert to alter solute and solvent movement in response to ABA signalling during dormancy cycling and up to the point of radicle emergence. Both TIP3 are induced by ABA but then respond differently to changing sensitivity to ABA with TIP3;1 responding positively in a dose dependent fashion to amplify the ABA response. TIP3;1 enables water transport (potentially restricting it when ABA sensitivity is high) but critically has potential to block fusion of the PSV preventing vacuolation (Lee et al., 2015) and therefore generation of turgor resulting in the lack of cell expansion observed in dormant seeds. This is consistent with our field observations that TIP3;1 expression exceeded that of TIP3;2 over winter during deep dormancy when ABA level was high. As dormancy declined to the shallow phase in early summer this difference was lost. At this point, ABA sensitivity decreases and seeds are increasingly sensitive to environmental signals that remove the final layer of dormancy. On receipt of such a signal (e.g., light), TIP3;2 may act in a dose dependent manner to reduce the impact of TIP3;1 enabling vacuolation to commence. As ABA sensitivity decreases TIP3;2 would likely increase solute uptake in tandem with increasing TIP3;1 activity to generate the turgor pressure required to drive germination. TIP4;1 may play a bet hedging role in response to the environment to modulate sensitivity. If dormancy is not alleviated, the balance between the TIP3 isoforms is reversed and dormancy increases.

In conclusion, we show that the TIP3 isoforms, rather than having redundant functions, have distinct and opposing roles in environmental sensing in seeds. We reveal the previously hidden role of TIP3 and TIP4;1 in regulating dormancy and germination in seeds exposed to temperature and water stress experienced by seeds dispersed under field conditions where they undergo dormancy cycling. The role extends through the continuum of the dormancy cycle that controls the timing of the completion of seed germination in the natural environment. This annual cycle is crucial in habitat selection, and success in competitive plant communities and so contributes to species fitness.

## AUTHOR CONTRIBUTIONS

S. F., L. F. and W. F. S. designed research; R. C., S. F. and M. F. performed research; S. F., W. F. S., and L. F. analysed data; and S. F., L. F. and W. F.‐S. wrote the paper.

## Supporting information


**Figure S1.** Seeds from a line co‐expressing TIP3;2‐mCherry (red) and TIP4;1‐YFP (green) under their native promoters were imaged by confocal microscopy, after seed coat removal, after testa rupture, allowing radicle emergence to complete germination. While TIP3;2 mCherry labels the vacuolar system, which is undergoing extensive remodelling upon germination, TIP4;1 is just becoming detectable and still labels the endoplasmic reticulum network as well as the tonoplast. Scale bar, 10 μm.

**Figure S2.** Characterisation of seed from mutant plant lines. **A**, map of the T‐DNA insertions in the indicated gene sequences. **B**, RT‐PCR analysis of relative TIP4;1 and tubulin (housekeeping gene) expression in the tip4;1 mutant and Col‐0 wild‐type. After 40 PCR cycles tip4;1 shows significantly reduced (but some residual) expression of the target gene. **C**, RT‐PCR analysis of relative TIP3 and ASAR1 (housekeeping gene) expression in the tip3 single and double mutants as well as Col‐0 wild‐type. After 28 cycles tip3;1 shows reduced expression of the target gene whereas tip3;2 is completely knocked out. **D**, Western blot analysis of GFP and TIP3 protein expression in Col‐0 wildtype, TIP3;2‐TIP3;2‐YFP (under native TIP3;2 promoter), tip3;1tip3;2 and its complemented line (tip3;1tip3;2::TIP3;2‐TIP3;2‐YFP). Western blot analysis of TIP3 protein expression in Col‐0 wild‐type and tip3 mutant lines showing significantly decreased expression of TIP3 in tip3;2 and tip3;1tip3;2. Lower panels: Coomassie Brilliant Blue staining to visualise protein loading. In the right hand panel, protein from tip3;1 tip3;2 was loaded at twice the concentration of the other samples.

**Table S1.** SALK line primers for checking homozygosity
**Table S2.** Primers used for QPCR of field samples.
**Table S3.** Correlations of the annual expression patterns of TIP3.1 and TIP3.2 genes and TIP3 protein levels with environmental signals and the expression patterns of a range of dormancy and germination related genes.

**Data S1.** Heat maps of aquaporin expression during Arabidopsis germination

**Data S2.** Identities of genes co‐expressed with the TIP3 isoforms and TIP4;1 in the endosperm and radicle

**Data S3.** Heat maps of aquaporin expression during dormancy cycling.
