# Supplementary figures and images for: Aquaporins influence seed dormancy and germination in response to stress
Source: Plant Cell Environ. 2019 May 9;42(8):2325–39. doi: 10.1111/pce.13561 (PMC6767449; doi:10.1111/pce.13561)

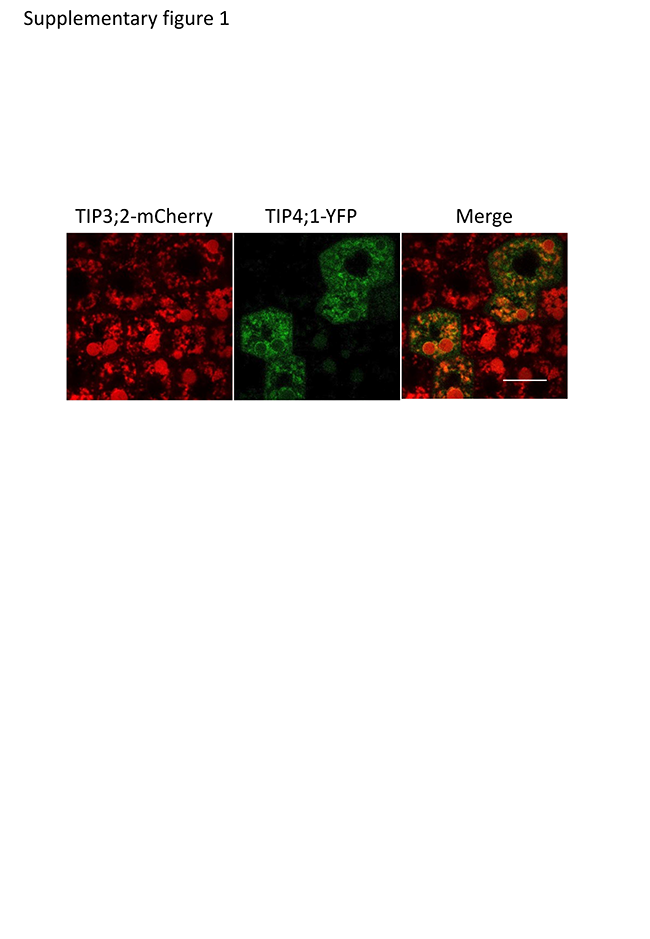

Supplement: Supplementary file 1 — Figure S1. Seeds from a line co‐expressing TIP3;2‐mCherry (red) and TIP4;1‐YFP (green) under their native promoters were imaged by confocal microscopy, after seed coat removal, after testa rupture, allowing radicle emergence to complete germination. While TIP3;2 mCherry labels the vacuolar system, which is undergoing extensive remodelling upon germination, TIP4;1 is just becoming detectable and still labels the endoplasmic reticulum network as well as the tonoplast. Scale bar, 10 μm. [file PCE-42-2325-s001.tif]

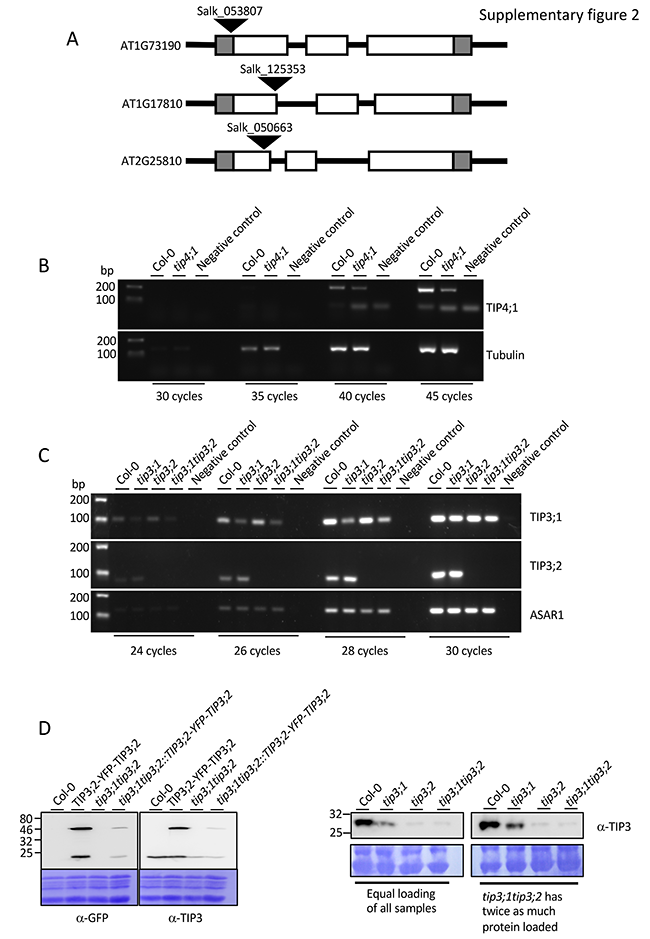

Supplement: Supplementary file 2 — Figure S2. Characterisation of seed from mutant plant lines. A, map of the T‐DNA insertions in the indicated gene sequences. B, RT‐PCR analysis of relative TIP4;1 and tubulin (housekeeping gene) expression in the tip4;1 mutant and Col‐0 wild‐type. After 40 PCR cycles tip4;1 shows significantly reduced (but some residual) expression of the target gene. C, RT‐PCR analysis of relative TIP3 and ASAR1 (housekeeping gene) expression in the tip3 single and double mutants as well as Col‐0 wild‐type. After 28 cycles tip3;1 shows reduced expression of the target gene whereas tip3;2 is completely knocked out. D, Western blot analysis of GFP and TIP3 protein expression in Col‐0 wildtype, TIP3;2‐TIP3;2‐YFP (under native TIP3;2 promoter), tip3;1tip3;2 and its complemented line (tip3;1tip3;2::TIP3;2‐TIP3;2‐YFP). Western blot analysis of TIP3 protein expression in Col‐0 wild‐type and tip3 mutant lines showing significantly decreased expression of TIP3 in tip3;2 and tip3;1tip3;2. Lower panels: Coomassie Brilliant Blue staining to visualise protein loading. In the right hand panel, protein from tip3;1 tip3;2 was loaded at twice the concentration of the other samples. [file PCE-42-2325-s002.tif]
