# Supplementary material for: Aquaporins influence seed dormancy and germination in response to stress
Source: Plant Cell Environ. 2019 May 9;42(8):2325–39. doi: 10.1111/pce.13561 (PMC6767449; doi:10.1111/pce.13561)
Supplement: Supplementary file 3 — Table S1. SALK line primers for checking homozygosity Table S2. Primers used for QPCR of field samples. Table S3. Correlations of the annual expression patterns of TIP3.1 and TIP3.2 genes and TIP3 protein levels with environmental signals and the expression patterns of a range of dormancy and germination related genes. [file PCE-42-2325-s003.docx]

**Supplemental data**

**Supplementary Data 1.** Heat maps of aquaporin expression during *Arabidopsis* germination

**Supplementary Data 2.** Identities of genes co-expressed with the TIP3 isoforms and TIP4;1 in the endosperm and radicle

**Supplementary Data 3.** Heat maps of aquaporin expression during dormancy cycling.

Table S1. SALK line primers for checking homozygosity

**Table S2.** Primers used for QPCR of field samples.

**Table S3.** Correlations of *TIP3* transcript profiles and TIP3 protein levels with environmental signals and dormancy and germination related genes during dormancy cycling in the field.

**Supplemental Figure S1. S1.** Visualising Expression of TIP3;2 and TIP4;1

**Supplemental Figure S2.** Characterisation of seed from mutant plant lines
