# Supplementary material for: Aquaporins influence seed dormancy and germination in response to stress
Source: Plant Cell Environ. 2019 May 9;42(8):2325–39. doi: 10.1111/pce.13561 (PMC6767449; doi:10.1111/pce.13561)

Supplemental data S1. Heat maps of aquaporin expression during *Arabidopsis* germination

Data was extracted from publically available microarray data sets. Data for expression in the radicle and micropylar endosperm comes from Dekkers et al. (2013) available from the Nottingham eFP browser (http://ssbvseed01.nottingham.ac.uk/efp_browser/efpWeb.cgi). Data for whole seed germination arrays is from Narsai et al. (2011) available from the Arabidopsis eFP browser (http://bar.utoronto.ca)

Dekkers BJ, Pearce S, van Bolderen-Veldkamp RP, Marshall A, Widera P, Gilbert J, Drost HG, Bassel GW, Muller K, King JR, et al. 2013. Transcriptional dynamics of two seed compartments with opposing roles in Arabidopsis seed germination. *Plant Physiology* **163**(1): 205-215.

Narsai R., Law S.R., Carrie C., Xu L & Whelan J. (2011) In depth temporal transcriptome profiling reveals a crucial developmental switch with roles for RNA processing and organelle metabolism that are essential for germination in *Arabidopsis thaliana*. *Plant physiology*, **157,** 1342-1362.

PIP1;1

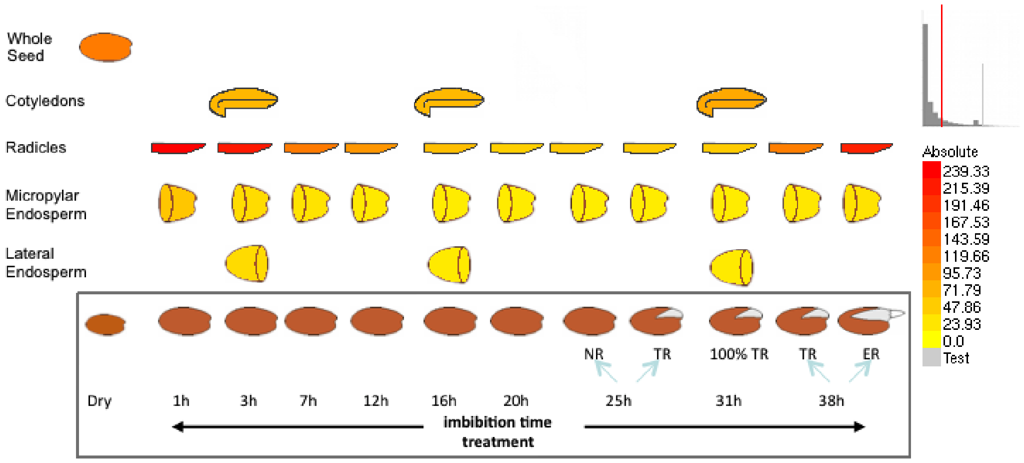

PIP1;2

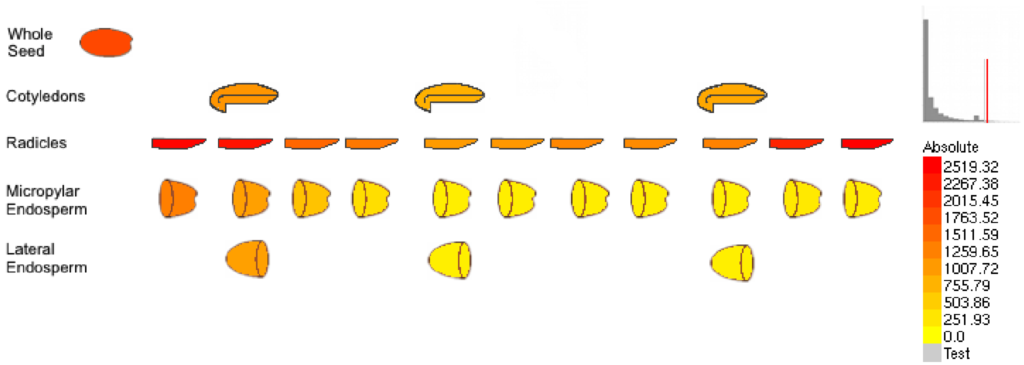

PIP1;3

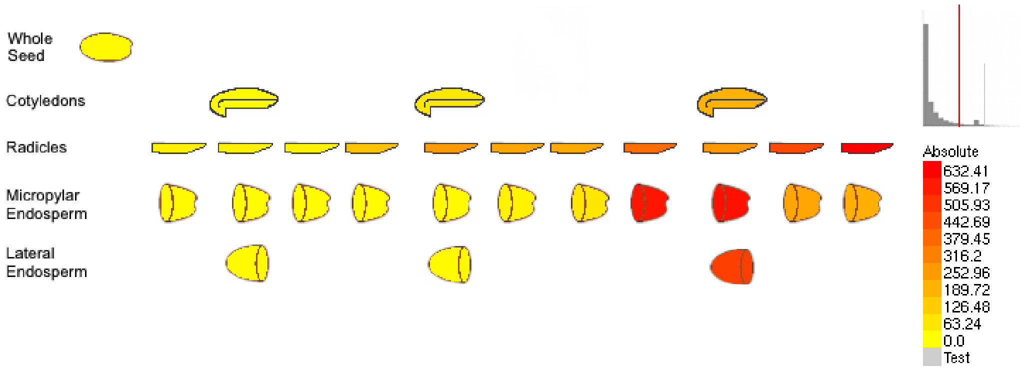

PIP1;4

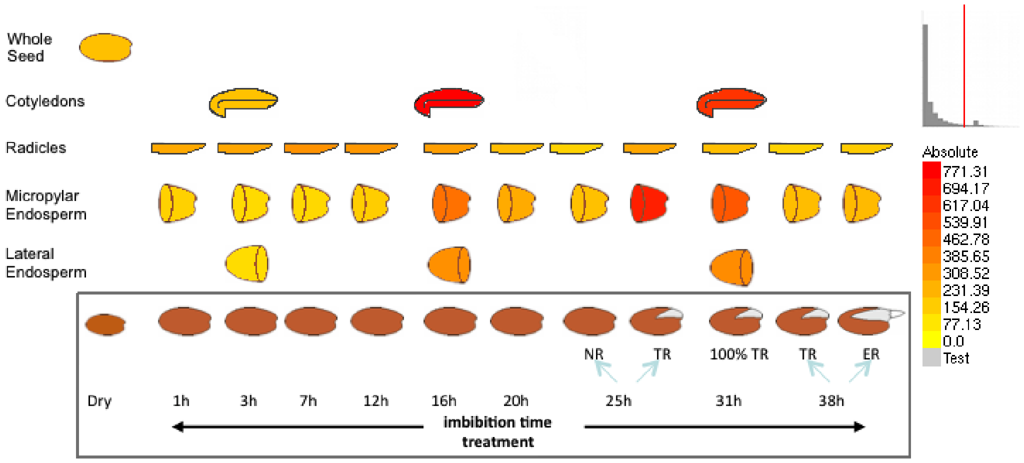

PIP1;5

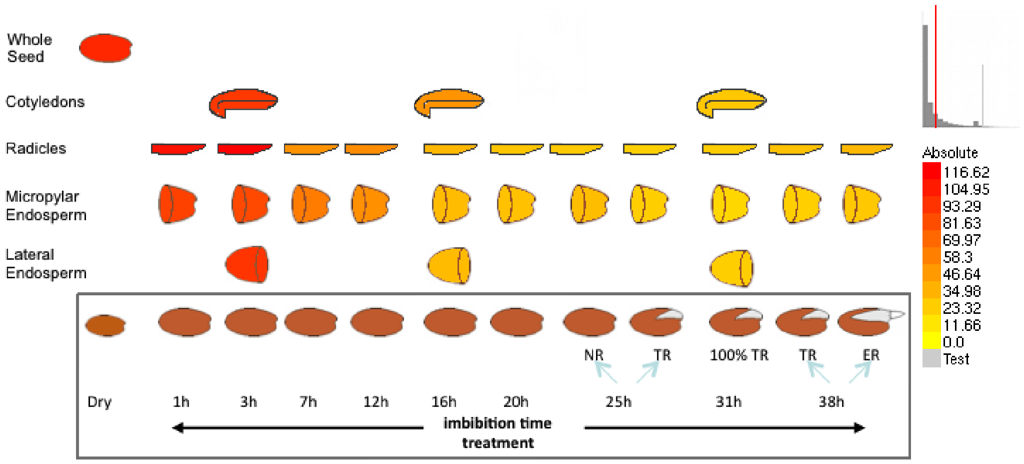

PIP2;1

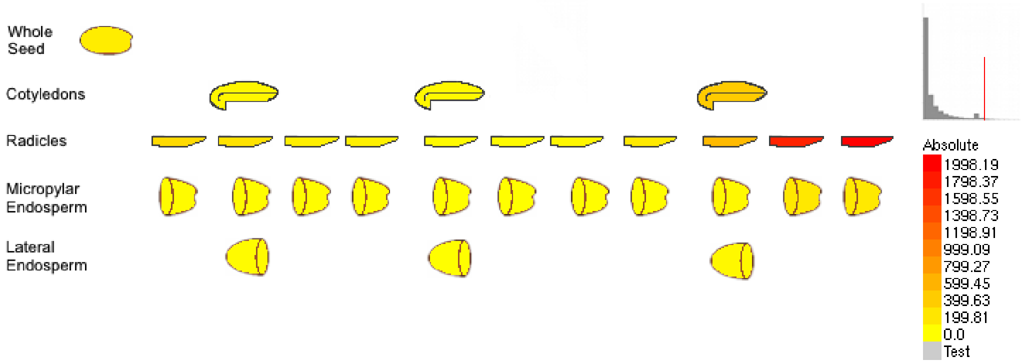

PIP2;5

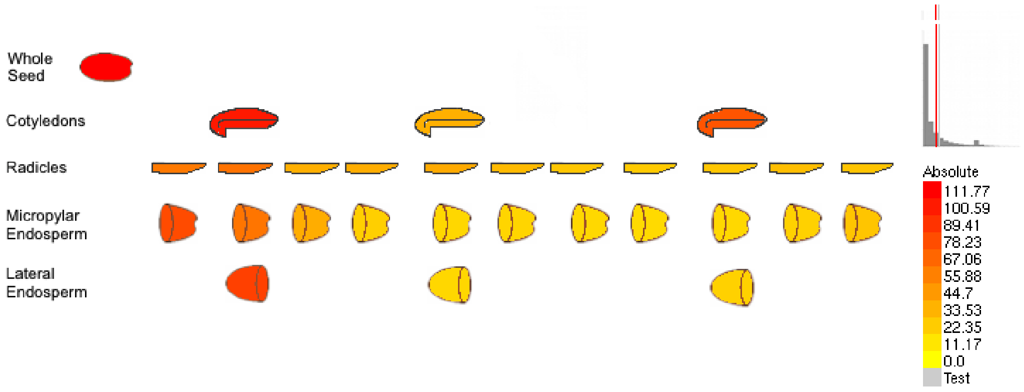

PIP2;6

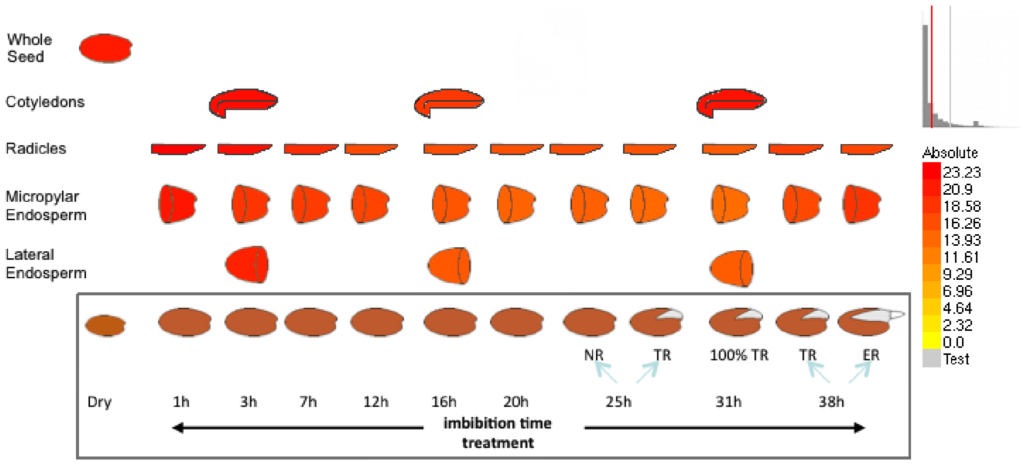

PIP2;7

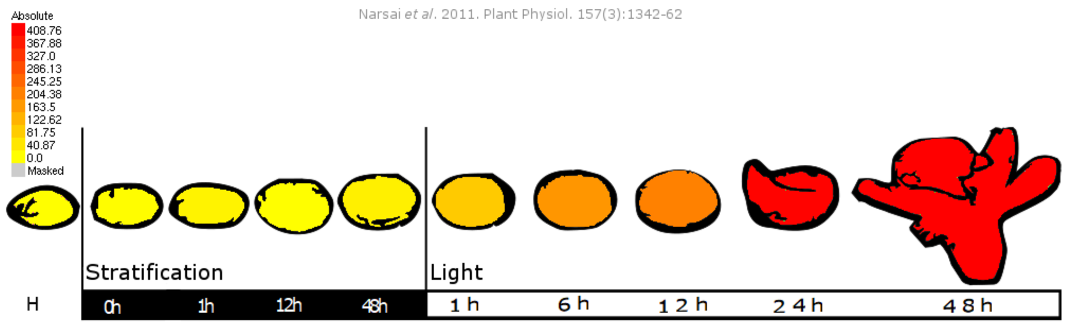

PIP2;8

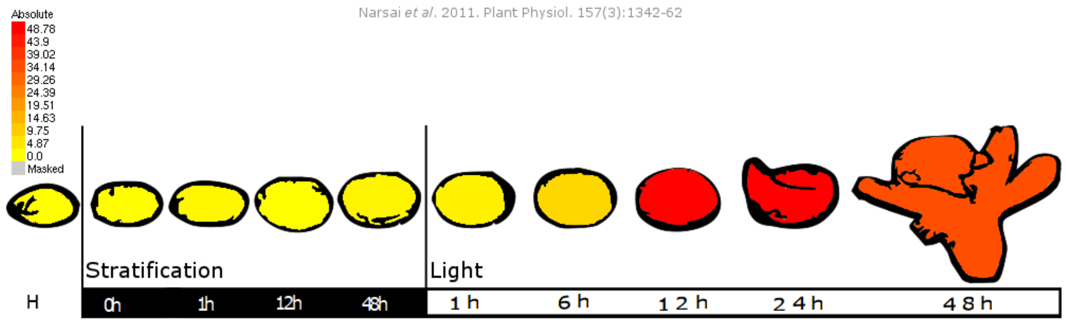

TIP1;1

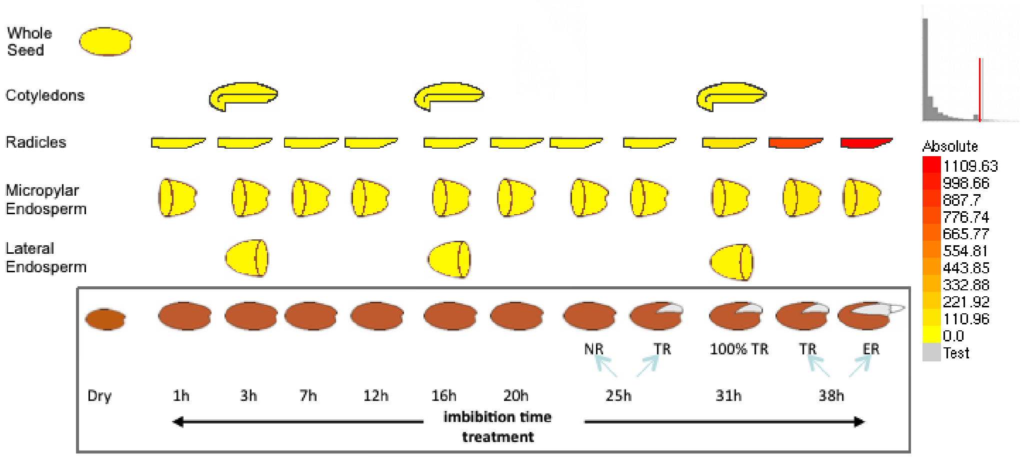

TIP1;2

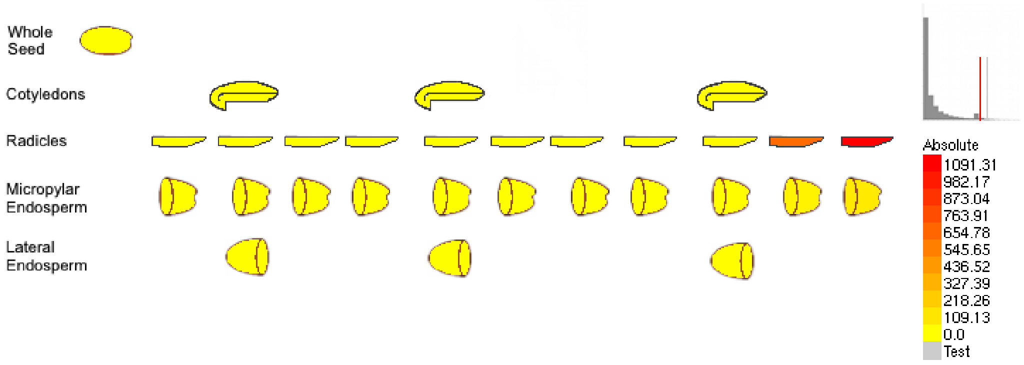

TIP1;3

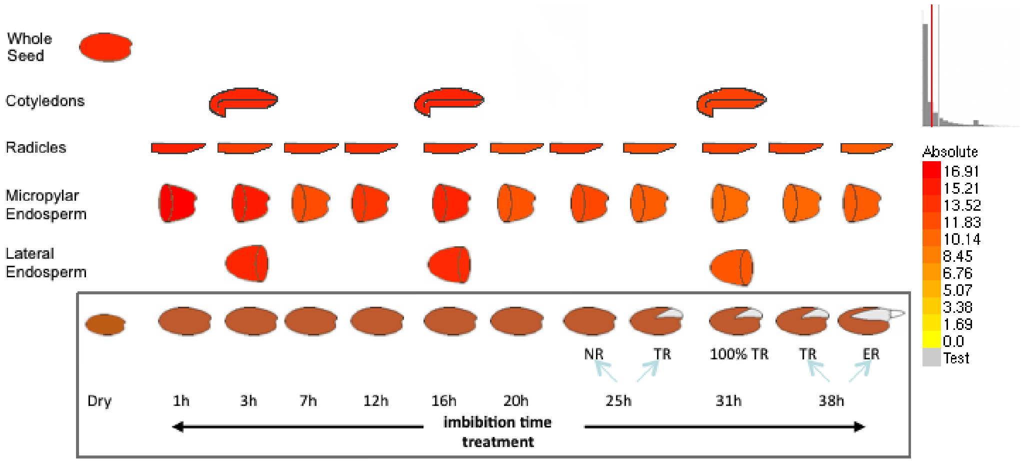

TIP2;1

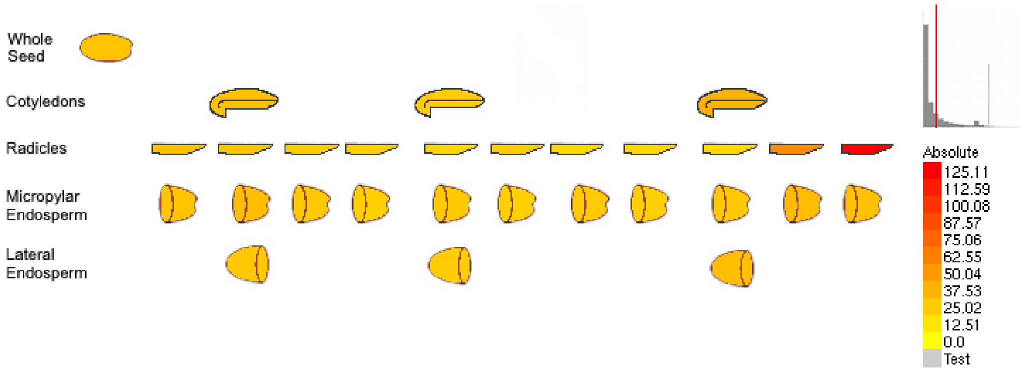

TIP2;3

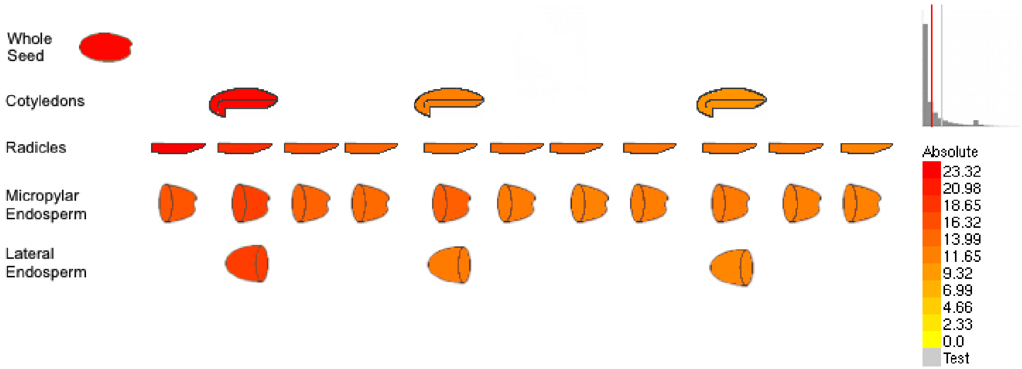

TIP3;1

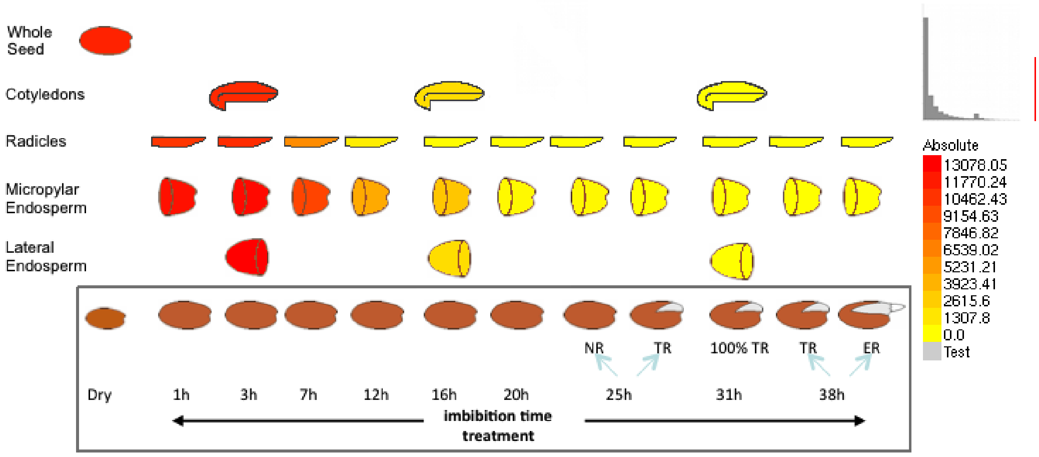

TIP3;2

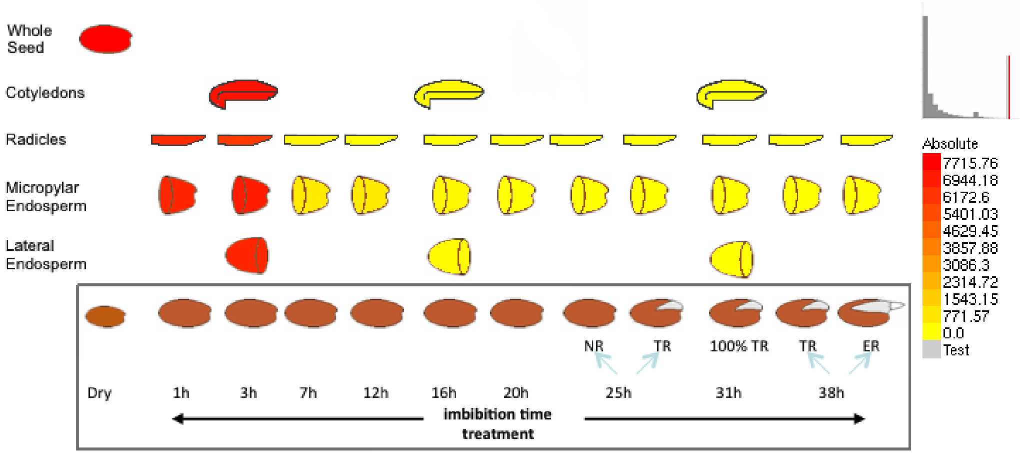

TIP4;1

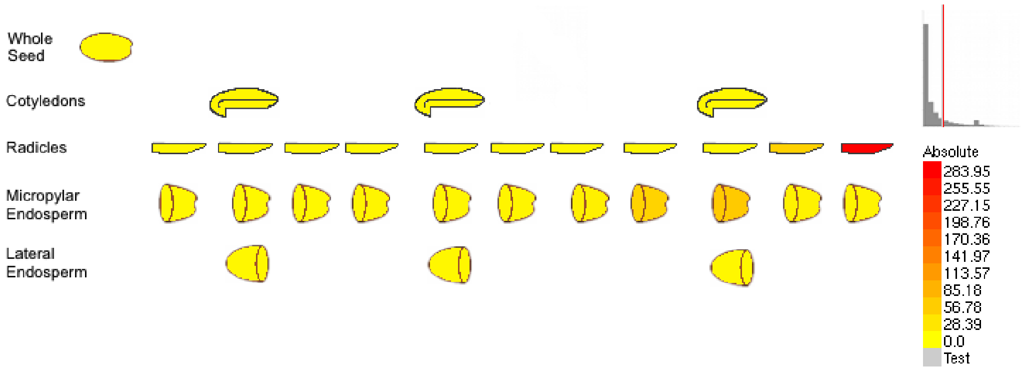

TIP5;1

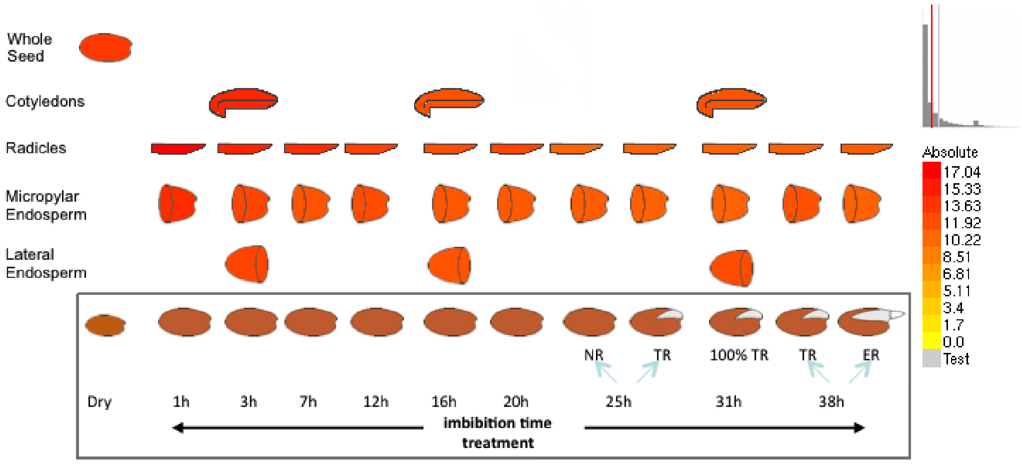

Narsai *et al*. 2011. Plant Physiol. 157(3):1342-62

NIP1;1

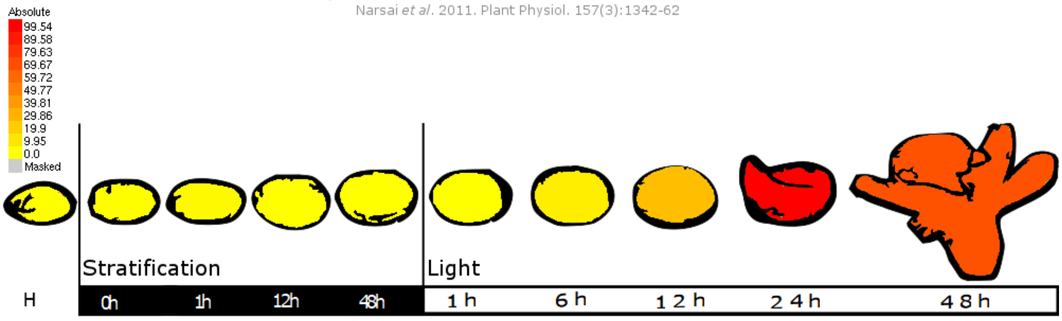

NIP1;2

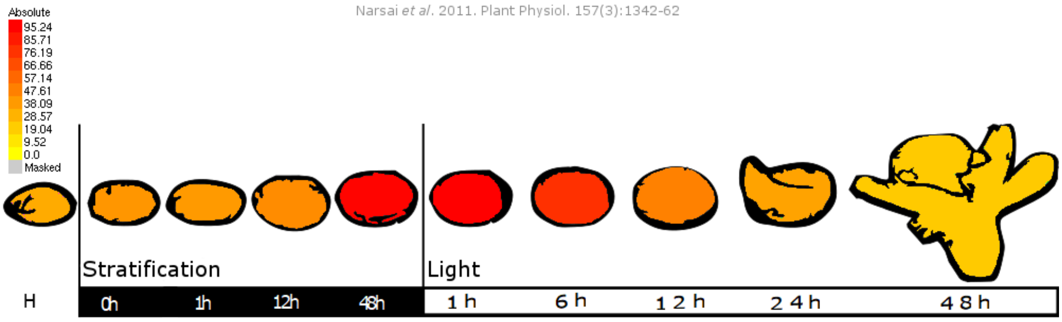

NIP2;1

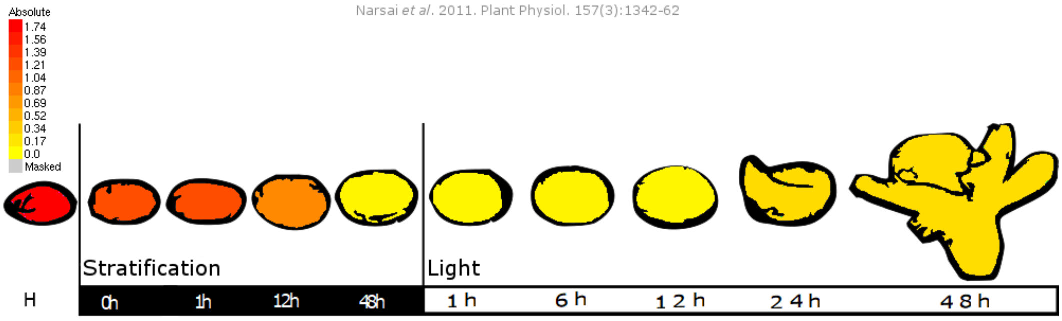

NIP3;1

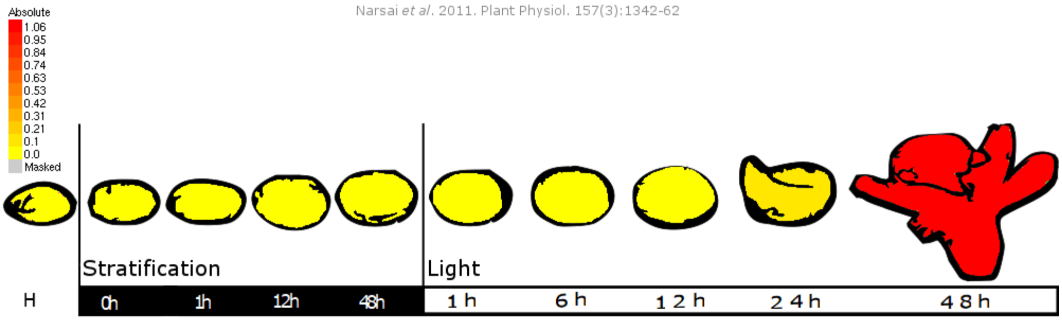

NIP4;1

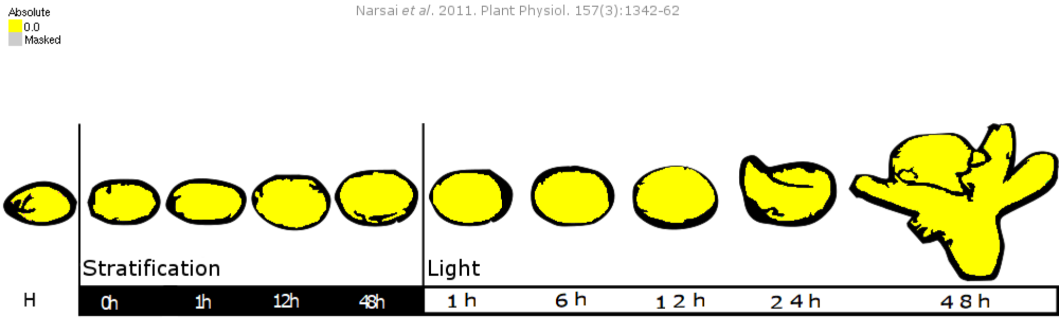

NIP4;2

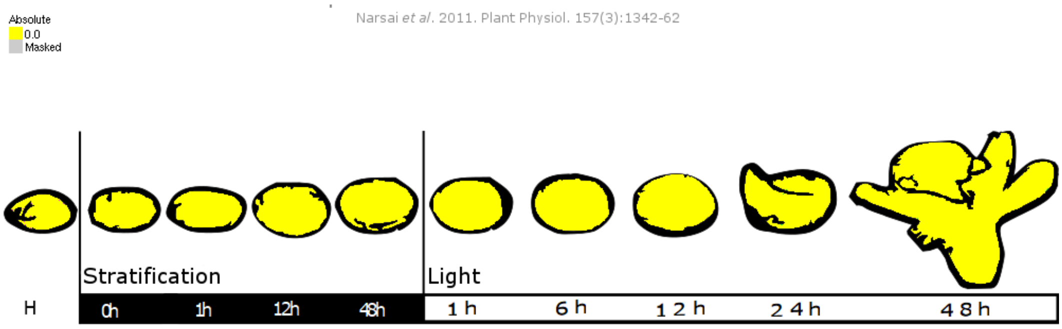

NIP5;1

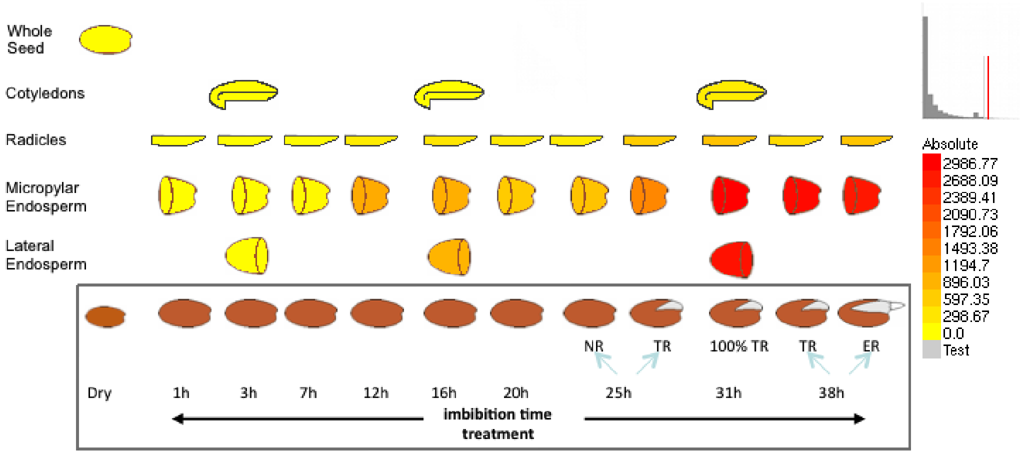

NIP6;1

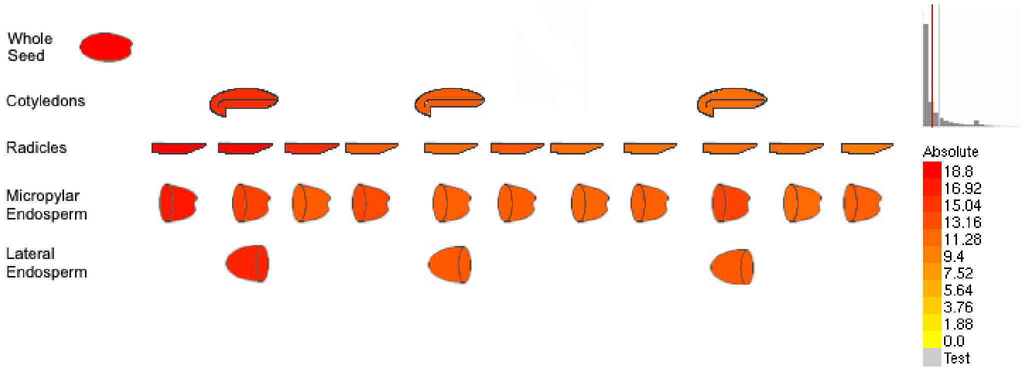

NIP7;1

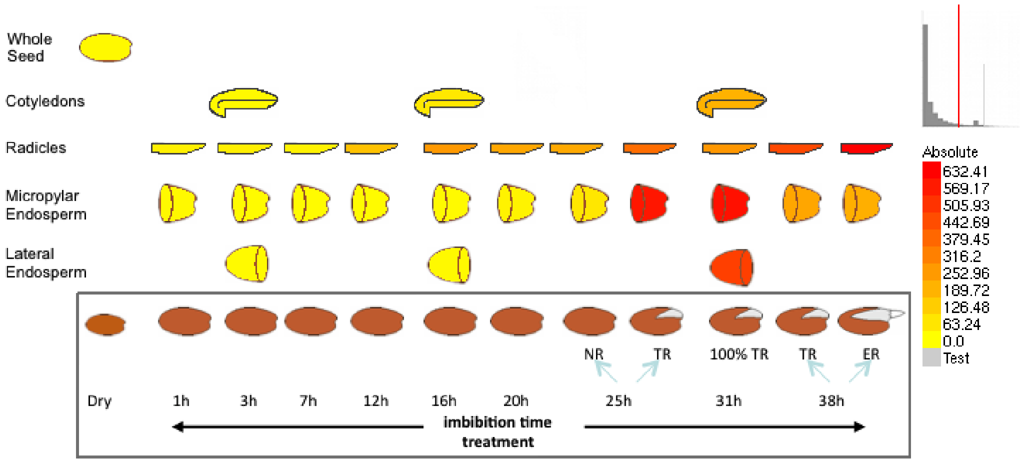

Supplement: Supplementary file 4 — Data S1. Heat maps of aquaporin expression during Arabidopsis germination [file PCE-42-2325-s004.pdf]
